# PR interval genome-wide association meta-analysis identifies 50 loci associated with atrial and atrioventricular electrical activity

**DOI:** 10.1038/s41467-018-04766-9

**Published:** 2018-07-25

**Authors:** Jessica van Setten, Jennifer A. Brody, Yalda Jamshidi, Brenton R. Swenson, Anne M. Butler, Harry Campbell, Fabiola M. Del Greco, Daniel S. Evans, Quince Gibson, Daniel F. Gudbjartsson, Kathleen F. Kerr, Bouwe P. Krijthe, Leo-Pekka Lyytikäinen, Christian Müller, Martina Müller-Nurasyid, Ilja M. Nolte, Sandosh Padmanabhan, Marylyn D. Ritchie, Antonietta Robino, Albert V. Smith, Maristella Steri, Toshiko Tanaka, Alexander Teumer, Stella Trompet, Sheila Ulivi, Niek Verweij, Xiaoyan Yin, David O. Arnar, Folkert W. Asselbergs, Joel S. Bader, John Barnard, Josh Bis, Stefan Blankenberg, Eric Boerwinkle, Yuki Bradford, Brendan M. Buckley, Mina K. Chung, Dana Crawford, Marcel den Hoed, Josh C. Denny, Anna F. Dominiczak, Georg B. Ehret, Mark Eijgelsheim, Patrick T. Ellinor, Stephan B. Felix, Oscar H. Franco, Lude Franke, Tamara B. Harris, Hilma Holm, Gandin Ilaria, Annamaria Iorio, Mika Kähönen, Ivana Kolcic, Jan A. Kors, Edward G. Lakatta, Lenore J. Launer, Honghuang Lin, Henry J. Lin, Ruth J. F. Loos, Steven A. Lubitz, Peter W. Macfarlane, Jared W. Magnani, Irene Mateo Leach, Thomas Meitinger, Braxton D. Mitchell, Thomas Munzel, George J. Papanicolaou, Annette Peters, Arne Pfeufer, Peter P. Pramstaller, Olli T. Raitakari, Jerome I. Rotter, Igor Rudan, Nilesh J. Samani, David Schlessinger, Claudia T. Silva Aldana, Moritz F. Sinner, Jonathan D. Smith, Harold Snieder, Elsayed Z. Soliman, Timothy D. Spector, David J. Stott, Konstantin Strauch, Kirill V. Tarasov, Unnur Thorsteinsdottir, Andre G. Uitterlinden, David R. Van Wagoner, Uwe Völker, Henry Völzke, Melanie Waldenberger, Harm Jan Westra, Philipp S. Wild, Tanja Zeller, Alvaro Alonso, Christy L. Avery, Stefania Bandinelli, Emelia J. Benjamin, Francesco Cucca, Marcus Dörr, Luigi Ferrucci, Paolo Gasparini, Vilmundur Gudnason, Caroline Hayward, Susan R. Heckbert, Andrew A. Hicks, J. Wouter Jukema, Stefan Kääb, Terho Lehtimäki, Yongmei Liu, Patricia B. Munroe, Afshin Parsa, Ozren Polasek, Bruce M. Psaty, Dan M. Roden, Renate B. Schnabel, Gianfranco Sinagra, Kari Stefansson, Bruno H. Stricker, Pim van der Harst, Cornelia M. van Duijn, James F. Wilson, Sina A. Gharib, Paul I. W. de Bakker, Aaron Isaacs, Dan E. Arking, Nona Sotoodehnia

**Affiliations:** 1 Department of Cardiology, University Medical Center Utrecht, University of Utrecht, Utrecht, 3584CX The Netherlands; 2 Department of Medicine, Cardiovascular Health Research Unit, University of Washington, Seattle, 98195 WA USA; 3 Cardiogenetics Lab, Molecular and Clinical Sciences Research Institute, St George’s University of London, London, SW17 0RE UK; 4 Institute for Public Health Genetics, School of Public Health, University of Washington, Seattle, 98195 WA USA; 5 Division of Infectious Diseases, Washington University School of Medicine, St. Louis, 63110 MO USA; 6 Centre for Global Health Research, Usher Institute of Population Health Sciences and Informatics, University of Edinburgh, Edinburgh, EH8 9YL UK; 7 Institute for Biomedicine, Eurac Research, Affiliated Institute of the University of Lübeck, Bolzano, 39100 Italy; 8 California Pacific Medical Center Research Institute, San Francisco, 94107 CA USA; 9 Department of Surgery, University of Alabama Birmingham Hospital, Birmingham, 35233 AL USA; 10 deCODE genetics/Amgen, Inc., Reykjavik, IS-101 Iceland; 11 School of Engineering and Natural Sciences, University of Iceland, Reykjavik, 101 Iceland; 12 Department of Biostatistics, School of Public Health, University of Washington, Seattle, 98195 WA USA; 13 Department of Epidemiology, Erasmus Medical Center, Rotterdam, 3015GD The Netherlands; 14 Department of Clinical Chemistry, Fimlab Laboratories, Tampere, 33520 Finland; 15 Department of Clinical Chemistry, Finnish Cardiovascular Research Center-Tampere, Faculty of Medicine and Life Sciences, University of Tampere, Tampere, 33014 Finland; 16 Department of General and Interventional Cardiology, University Heart Center Hamburg-Eppendorf, Hamburg, 20251 Germany; 17 DZHK (German Center for Cardiovascular Research) Partner Site, Hamburg/Kiel/Lübeck, Germany; 18 Institute of Genetic Epidemiology, Helmholtz Zentrum München-German Research Center for Environmental Health, Neuherberg, D-85764 Germany; 19 Department of Internal Medicine I (Cardiology), Hospital of the Ludwig-Maximilians-University (LMU) Munich, Munich, 81377 Germany; 20 DZHK (German Centre for Cardiovascular Research), Partner Site Munich Heart Alliance, Munich, 80802 Germany; 21 Department of Epidemiology, University of Groningen, University Medical Center Groningen, Groningen, 9713GZ The Netherlands; 22 Institute of Cardiovascular and Medical Sciences, College of Medical, Veterinary and Life Sciences, University of Glasgow, Glasgow, G12 8TA Scotland UK; 23 Department of Genetics, University of Pennsylvania, Philadelphia, PA 19104 PA USA; 24 Institute for Maternal and Child Health—IRCCS “Burlo Garofolo”, Trieste, 34137 Italy; 25 Icelandic Heart Association, Kopavogur, IS-201 Iceland; 26 Faculty of Medicine, University of Iceland, Reykjavik, 101 Iceland; 27 Department of Biostatistics, University of Michigan, Ann Arbor, 48109 MI USA; 28 Istituto di Ricerca Genetica e Biomedica, Consiglio Nazionale delle Ricerche (CNR), Monserrato, 00185 Cagliari Italy; 29 Translational Gerontology Branch, NIA, Baltimore, 20892 MD USA; 30 Institute for Community Medicine, University Medicine Greifswald, Greifswald, 17489 Germany; 31 DZHK (German Centre for Cardiovascular Research), Partner Site Greifswald, 17475 Greifswald, Germany; 32 Department of Cardiology, Leiden University Medical Center, Leiden, 2300RC The Netherlands; 33 Department of Gerontology and Geriatrics, Leiden University Medical Center, Leiden, 2300RC The Netherlands; 34 Department of Cardiology, University Medical Center Groningen, University of Groningen, Groningen, 7913GZ The Netherlands; 35 Department of Medicine, Boston University School of Medicine, Boston, 02118 MA USA; 36 Department of Medicine, Landspitali University Hospital, Reykjavik, 101 Iceland; 37 Durrer Center for Cardiogenetic Research, Netherlands Heart Institute, Utrecht, The Netherlands; 38 Institute of Cardiovascular Science, Faculty of Population Health Sciences, University College London, London, WC1E 6BT UK; 39 Farr Institute of Health Informatics Research and Institute of Health Informatics, University College London, London, WC1E 6BT London United Kingdom; 40 Department of Biomedical Engineering, Johns Hopkins University, Baltimore, 21218 MD USA; 41 Department of Quantitative Health Sciences, Lerner Research Institute, Cleveland Clinic, Cleveland, 44195 OH USA; 42 Human Genetics Center, University of Texas Health Science Center at Houston, Houston, 77030 TX USA; 43 Department of Pharmacology and Therapeutics, University College Cork, Cork, T12 K8AF Ireland; 44 Department of Cardiovascular Medicine, Heart and Vascular Institute, Cleveland Clinic, Cleveland, 44195 OH USA; 45 Department of Molecular Cardiology, Lerner Research Institute, Cleveland Clinic, Cleveland, 44195 OH USA; 46 Institute for Computational Biology, Department of Population and Quantitative Health Sciences, Case Western Reserve University, Cleveland, 44106 OH USA; 47 Department of Immunology, Genetics and Pathology, Uppsala University, Uppsala, SE-751 05 Sweden; 48 Science for Life Laboratory, Uppsala University, Uppsala, SE-751 05 Sweden; 49 Biomedical Informatics and Medicine, Vanderbilt University, Nashville, 37235 TN USA; 50 Center for Complex Disease Genomics, McKusick-Nathans Institute of Genetic Medicine, Johns Hopkins University School of Medicine, Baltimore, 21287 MD USA; 51 Department of Nephrology, University Medical Center Groningen, Groningen, 7913GZ The Netherlands; 52 Program in Medical and Population Genetics, The Broad Institute of MIT and Harvard, Cambridge, 02142 MA USA; 53 Cardiovascular Research Center, Massachusetts General Hospital, Boston, 02114 MA USA; 54 Cardiac Arrhythmia Service, Massachusetts General Hospital, Boston, 02114 MA USA; 55 Department of Internal Medicine B, University Medicine Greifswald, Greifswald, 17489 Germany; 56 Department of Genetics, University of Groningen, University Medical Center Groningen, Groningen, 7913GZ The Netherlands; 57 Laboratory of Epidemiology and Population Sciences, National Institute on Aging, Intramural Research Program, National Institutes of Health, Bethesda, 20892 MD USA; 58 Department of Medical Sciences, University of Trieste, Trieste, 34127 Italy; 59 Cardiovascular Department, “Ospedali Riuniti and University of Trieste”, Trieste, 34124 Italy; 60 Department of Clinical Physiology, Tampere University Hospital, Tampere, 33521 Finland; 61 Department of Clinical Physiology, Finnish Cardiovascular Research Center-Tampere, Faculty of Medicine and Life Sciences, University of Tampere, Tampere, 33014 Finland; 62 Faculty of Medicine, University of Split, Split, 21000 Croatia; 63 Department of Medical Informatics, Erasmus University Medical Center, Rotterdam, 3015GD The Netherlands; 64 Laboratory of Cardiovascular Science, National Institute on Aging, National Institutes of Health, Baltimore, 20892 MD USA; 65 The Institute for Translational Genomics and Population Sciences, Department of Pediatrics, Los Angeles Biomedical Research Institute at Harbor-UCLA Medical Center, Torrance, 90502 CA USA; 66 The Charles Bronfman Institute for Personalized Medicine, Icahn School of Medicine at Mount Sinai, New York, 10029 NY USA; 67 The Mindich Child health and Development Institute, Icahn School of Medicine at Mount Sinai, New York, 10029 NY USA; 68 Institute of Health and Wellbeing, College of Medical, Veterinary and Life Sciences, University of Glasgow, Glasgow, G12 8QQ UK; 69 Department of Medicine, Division of Cardiology, University of Pittsburgh Medical Center Heart and Vascular Institute, University of Pittsburgh, Pittsburgh, 15260 PA USA; 70 Institute of Human Genetics, Helmholtz Zentrum München-German Research Center for Environmental Health, Neuherberg, 85764 Germany; 71 Institute of Human Genetics, Klinikum rechts der Isar, Technische Universität München, Munich, 81675 Germany; 72 Department of Medicine, University of Maryland School of Medicine, Geriatrics Research and Education Clinical Center, Baltimore VA Medical Center, Baltimore, 21201 MD USA; 73 Center for Cardiology, University Medical Center of the Johannes Gutenberg-University Mainz, Mainz, 55131 Germany; 74 DZHK (German Center for Cardiovascular Research), Partner Site Rhine-Main, Mainz, 55131 Germany; 75 Center for Translational Vascular Biology (CTVB), University Medical Center of the Johannes Gutenberg-University Mainz, Mainz, 55131 Germany; 76 Division of Cardiovascular Sciences, National Heart, Lung, and Blood Institute, NIH, Bethesda, 20892 MD USA; 77 Institute of Epidemiology, Helmholtz Zentrum München-German Research Center for Environmental Health, Neuherberg, 85764 Germany; 78 German Center for Diabetes Research, Neuherberg, 85764 Germany; 79 MVZ für Molekulardiagnostik, Munich, 81543 Germany; 80 Department of Neurology, Central Hospital, Bolzano, 39100 Italy; 81 Department of Clinical Physiology and Nuclear Medicine, Turku University Hospital, Turku, 20521 Finland; 82 Research Centre of Applied and Preventive Cardiovascular Medicine, University of Turku, Turku, 20014 Finland; 83 Departments of Medicine and Pediatrics, The Institute for Translational Genomics and Population Sciences, Los Angeles Biomedical Research Institute at Harbor-UCLA Medical Center, Torrance, 90502 CA USA; 84 Department of Cardiovascular Sciences, University of Leicester, Leicester, LE1 7RH UK; 85 NIHR Leicester Biomedical Research Centre, Leicester, LE3 9QD UK; 86 Laboratory of Genetics and Genomics, National Institute on Aging, National Institute of Health, Baltimore, 20892 MD USA; 87 Genetic Epidemiology Unit, Department of Epidemiology, Erasmus University Medical Center, Rotterdam, 3015GD The Netherlands; 88 Institute of Translational Medicine—IMT, Center For Research in Genetics and Genomics-CIGGUR, GENIUROS Research Group, School of Medicine and Health Sciences, Universidad del Rosario, Bogotá, Cl. 12c #6-25 Colombia; 89 Department of Cellular and Molecular Medicine Biology, Lerner Research Institute, Cleveland Clinic, Cleveland, 44195 OH USA; 90 Epidemiological Cardiology Research Center, Wake Forest School of Medicine, Winston-Salem, 27101 NC USA; 91 Department of Twin Research and Genetic Epidemiology, St Thomas Hospital, King’s College London, London, WC2R 2LS UK; 92 Chair of Genetic Epidemiology, IBE, Faculty of Medicine, LMU Munich, Munich, 81377 Germany; 93 Faculty of Medicine, University of Iceland, Reykjavik, 101 Iceland; 94 Department of Internal Medicine, Erasmus Medical Center, Rotterdam, 3015GD The Netherlands; 95 Interfaculty Institute for Genetics and Functional Genomics, University Medicine Greifswald, Greifswald, 17489 Germany; 96 Research Unit of Molecular Epidemiology, Helmholtz Zentrum München-German Research Center for Environmental Health, Neuherberg, 85764 Germany; 97 Preventive Cardiology and Preventive Medicine, Center for Cardiology, University Medical Center of the Johannes Gutenberg-University Mainz, Mainz, 55131 Germany; 98 Center for Thrombosis and Hemostasis, University Medical Center of the Johannes Gutenberg-University Mainz, Mainz, 55131 Germany; 99 Department of Epidemiology, Rollins School of Public Health, Emory University, Atlanta, 30322 GA USA; 100 Department of Epidemiology and Carolina Population Center, University of North Carolina at Chapel Hill, Chapel Hill, NC 27514 USA; 101 Geriatric Unit, Azienda Sanitaria Firenze (ASF), Florence, 50122 Italy; 102 Medical Research Council Human Genetics Unit, Institute of Genetics and Molecular Medicine, University of Edinburgh, Edinburgh, EH8 9YL UK; 103 Cardiovascular Health Research Unit and the Department of Epidemiology, University of Washington, Seattle, 98195 WA USA; 104 Einthoven Laboratory for Experimental Vascular Medicine, Leiden University Medical Center, Leiden, 2300RC The Netherlands; 105 Interuniversity Cardiology Institute of the Netherlands, Utrecht, 3511EP The Netherlands; 106 Department of Epidemiology and Prevention, Division of Public Health Sciences, Wake Forest University, Winston-Salem, 27101 NC USA; 107 Clinical Pharmacology, William Harvey Research Institute, Queen Mary University of London, London, E1 4NS UK; 108 NIHR Biomedical Research Centre at Barts, Barts Health NHS Trust and Queen Mary University of London, London, E1 4NS UK; 109 Department of Medicine, University of Maryland School of Medicine and Baltimore VA Medical Center, Baltimore, 21201 MD USA; 110 Psychiatric hospital “Sveti Ivan”, Zagreb, 10000 Croatia; 111 Gen-info Ltd., Zagreb, 10000 Croatia; 112 Cardiovascular Health Research Unit, Departments of Medicine, Epidemiology and Health Services, University of Washington, Seattle, 98195 WA USA; 113 Kaiser Permanente Washington Health Research Institute, Seattle, 98101 WA USA; 114 Medicine, Pharmacology, and Biomedical Informatics, Vanderbilt University, Nashville, 37235 TN USA; 115 Department of Internal Medicine, Erasmus Medical Center, Rotterdam, 3015GD The Netherlands; 116 Inspectorate for Health Care, The Hague, 2511VX The Netherlands; 117 University of Groningen, University Medical Center Groningen, Department of Genetics, Groningen, 7913GZ The Netherlands; 118 Durrer Center for Cardiogenetic Research, ICIN-Netherlands Heart Institute, Utrecht, 3511EP The Netherlands; 119 Cardiovascular Health Research Unit, Division of Pulmonary Critical Care and Sleep Medicine, Computational Medicine Core at Center for Lung Biology, Department of Medicine, University of Washington, Seattle, 98195 WA USA; 120 Department of Genetics, Center for Molecular Medicine, University Medical Center Utrecht, Utrecht, 3584CX The Netherlands; 121 Department of Epidemiology, Julius Center for Health Sciences and Primary Care, University Medical Center Utrecht, Utrecht, 3584CX The Netherlands; 122 CARIM School for Cardiovascular Diseases, Maastricht, 6229ER The Netherlands; 123 Center for Systems Biology (MaCSBio), Maastricht University, Maastricht, 6229ER The Netherlands; 124 Department of Biochemistry, Maastricht University, Maastricht, 6229ER The Netherlands; 125 McKusick-Nathans Institute of Genetic Medicine, Johns Hopkins University School of Medicine, Baltimore, 21287 MD USA; 126 Departments of Medicine and Epidemiology, Cardiovascular Health Research Unit, Division of Cardiology, University of Washington, Seattle, 98101 WA USA

## Abstract

Electrocardiographic PR interval measures atrio-ventricular depolarization and conduction, and abnormal PR interval is a risk factor for atrial fibrillation and heart block. Our genome-wide association study of over 92,000 European-descent individuals identifies 44 PR interval loci (34 novel). Examination of these loci reveals known and previously not-yet-reported biological processes involved in cardiac atrial electrical activity. Genes in these loci are over-represented in cardiac disease processes including heart block and atrial fibrillation. Variants in over half of the 44 loci were associated with atrial or blood transcript expression levels, or were in high linkage disequilibrium with missense variants. Six additional loci were identified either by meta-analysis of ~105,000 African and European-descent individuals and/or by pleiotropic analyses combining PR interval with heart rate, QRS interval, and atrial fibrillation. These findings implicate developmental pathways, and identify transcription factors, ion-channel genes, and cell-junction/cell-signaling proteins in atrio-ventricular conduction, identifying potential targets for drug development.

## Abstract (web summary)

Abnormal PR interval duration is associated with risk for atrial fibrillation and heart block. Here, van Setten et al. identify 44 PR interval loci in a genome-wide association study of over 92,000 individuals and find genetic overlap with QRS duration, heart rate and atrial fibrillation.

## Introduction

The PR interval on the surface electrocardiogram reflects atrial and atrioventricular node myocyte depolarization and conduction. Abnormalities in PR interval duration are associated with increased risk of atrial fibrillation (AF), which carries a substantial risk of morbidity and mortality, and with cardiac conduction defects and heart block, conditions that often necessitate pacemaker implantation^1^. Understanding the molecular mechanisms affecting the PR interval may provide insights into cardiac electrical disease processes, and identify potential drug targets for prevention and treatment of AF and conduction disease.

Twin and family studies suggest that the heritability of PR interval is between 40 and 60%^2^. Prior genome-wide association studies (GWAS) in up to 30,000 individuals have identified ten loci associated with PR interval among European-descent individuals^3, 4^. The key motivation for the present study is to extend the biological and clinical insights derived from GWAS data by utilizing the largest sample size to detect novel PR loci genome-wide. We further increase power by performing trans-ethnic meta-analyses. To gain additional biological and clinical insights, we test for pleiotropy with other clinically relevant phenotypes. We examine the biological and functional relevance of identified associations to gain insights into molecular processes underlying clinically important phenotypes.

Our GWAS of over 92,000 European-descent individuals identifies 44 loci (34 novel) associated with PR interval. Examination of the 44 loci revealed known and novel biological processes involved in cardiac atrial electrical activity, including cardiac sodium channels, transcription factors involved in cardiac development, and sarcomeric-related proteins. Ten of the 61 non-redundant variants in these 44 loci are in high linkage disequilibrium (LD) with one or more missense variants. More than half of the index variants influence transcript expression levels as measured in the atria or in blood, with the regulation of certain genes found only in atrial tissue. Indeed, cardiac regulatory regions of the genome as measured by cardiac DNA hypersensitivity sites are enriched for variants associated with PR interval, compared to non-cardiac regulatory regions. Genes in the 44 loci are highly over-represented in a number of disease processes, including sick sinus syndrome, heart block, and AF. This motivated us to perform pleiotropic analyses, where we jointly analyze the phenotypes of PR–heart rate; PR–QRS interval (a measure of ventricular conduction); and PR–AF, and identify an additional three new pleiotropic loci. More than half of the single nucleotide polymorphisms (SNPs) identified show evidence of pleiotropy with other electrophysiologic phenotypes; SNPs that influence atrial conduction also influence ventricular conduction, atrial arrhythmias, and/or heart rate. Trans-ethnic analyses suggest that the majority of the associations derived from European-descent population are also present in African-American population. Meta-analysis examining over 100,000 individuals of African and European descent identifies five novel PR loci (two of which are also identified by pleiotropic analyses). These findings underscore the power of GWAS to extend knowledge of the molecular underpinnings of clinical processes.

## Results

### PR interval meta-analysis of genome-wide association studies

**Fig. 1:**
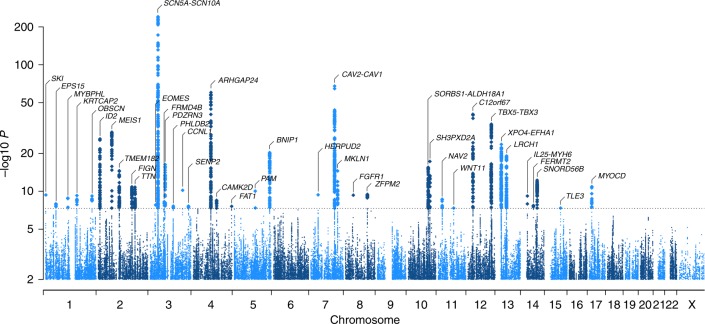
Genome-wide results of PR interval in 92,340 individuals of European descent. 2.8 million SNPs were tested for association with PR interval in 31 cohorts. The Manhattan plot shows the meta-analysis association results: 44 independent loci (labeled) are associated at the genome-wide significance level of *P* ≤ 5 × 10^−8^, as marked by the dashed line

**Fig. 2:**
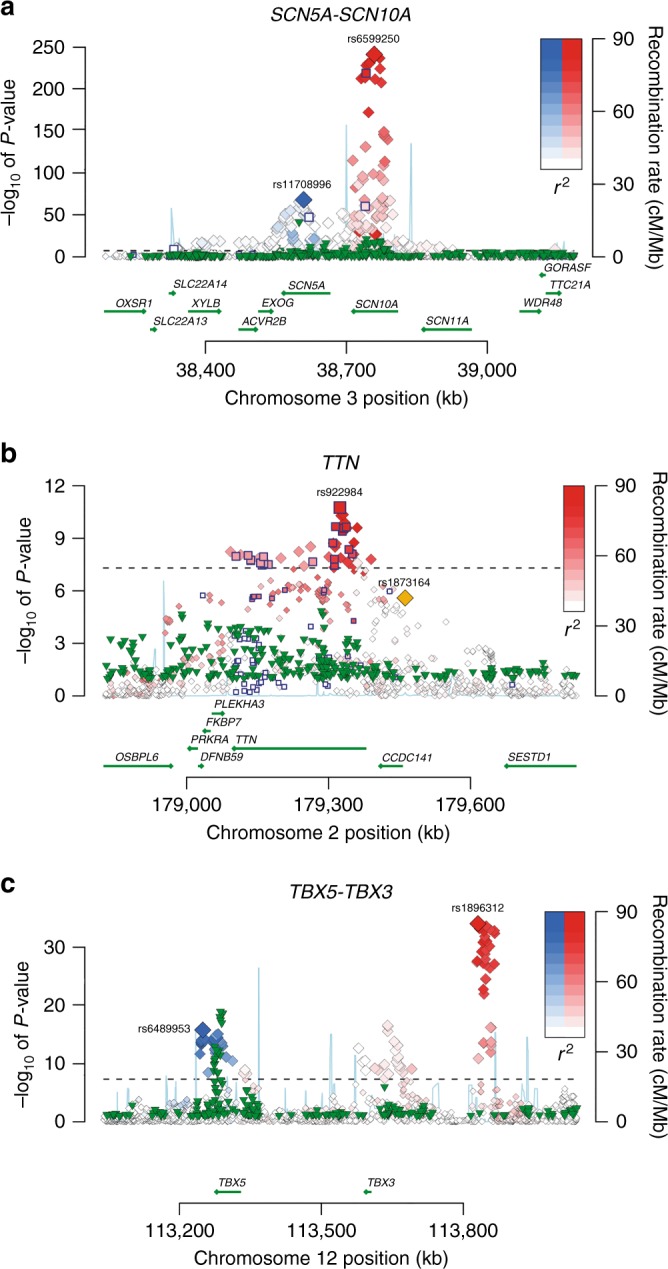
Regional association plots of specific loci associated with PR interval. Each SNP is plotted with respect to its chromosomal location (*x* axis) and its *P* value (*y* axis on the left). The blue line indicates the recombination rate (*y* axis on the right) at that region of the chromosome. Blue outlined squares mark non-synonymous SNPs. Green triangles depict association results of the African Americans meta-analysis, only SNPs with *P* < 0.1 are shown. **a** Locus 2 and 3 (*SCN10A*–*SCN5A*) on chromosome 3. The index SNPs for the two genes are named with their rs-numbers and highlighted with two different colors (blue and red). Other SNPs in linkage disequilibrium with the index SNP are denoted in the same color; color saturation indicates the degree of correlation with the index SNP. **b** Locus 19 (*TTN*) on chromosome 2; and **c** Locus 9 and 10 (*TBX5*–*TBX3*) on chromosome 12

We meta-analyzed ~2.7 million SNPs from GWAS data on 92,340 individuals of European ancestry from 31 studies (Supplementary Data 1 and 2) for association with PR interval using an additive genetic model. A total of 1601 SNPs mapping to 44 loci (of which 34 were novel in Europeans) reached genome-wide significance (*P* ≤ 5 × 10^−8^) (Fig. 1, Tables 1 and 2, Supplementary Figures 1 and 2). The genomic inflation factor lambda was 1.11 and LD score regression^5^ showed that that the inflation of the test statistic was mainly caused by true polygenicity (Supplementary Fig. 3). Using a Bayesian locus-based test of association (GWiS)^6^, we identified 61 non-redundant signals in the 44 loci (listed in Supplementary Data 4). For example, the top locus on chromosome 3, a known cardiac conduction locus mapping to the two cardiac sodium channel genes *SCN5A* and *SCN10A*^3, 4, 7, 8^, had seven non-redundant signals associated with PR interval (Fig. 2a).

**Table 1:** Description of PR loci previously identified by GWAS among those of European descent

European ancestry index SNPs in previously GWAS identified PR loci									Non-red SNPs	AA PR	Missense	DHS	Cardiac eQTL
Locus	SNP	Chr	Closest gene	CA	CAF	Beta (ms)	SE (ms)	*P*-value	*n*	*P* < 0.05	*r*^2^ > 0.8	*r*^2^ > 0.8	FDR < 0.05
**1**	rs4430933	2	*MEIS1*	A	0.39	1.3	0.11	5.06E−30	1	YES	–	YES	*MEIS1*
**2**	rs6599250	3	*SCN10A*	T	0.41	3.8	0.11	4.42E−242	2	YES	*SCN10A*	YES	*SCN10A & SCN5A*
**3**	rs11708996	3	*SCN5A*	C	0.15	3.1	0.18	1.06E−68	5	YES	*SCN5A*	YES	–
**4**	rs343849	4	*ARHGAP24*	A	0.3	−2.1	0.13	3.12E−61	1	YES	–	YES	–
**5**	rs255292	5	*BNIP1/NKX2-5*	C	0.42	−1.1	0.12	5.99E−21	1	YES	–	YES	*BNIP1*
**6**	rs3807989	7	*CAV1/CAV2*	A	0.41	2	0.12	8.65E−69	1	YES	–	YES	*CAV1*
**7**	rs652673	11	*WNT11*	C	0.22	−0.8	0.15	4.41E−08	1	–	–	–	–
**8**	rs17287293	12	*C12orf67/SOX5*	G	0.15	−2.2	0.16	2.33E−41	1	YES	–	–	–
**9**	rs1896312	12	*TBX3*	C	0.29	1.6	0.13	1.16E−34	4	–	–	–	–
**10**	rs6489953	12	*TBX5*	C	0.17	1.2	0.15	1.94E−16	2	–	–	–	–

For each of the 10 loci, we list the number of non-redundant signals, whether this locus is nominally significant in African Americans, if missense SNPs are in LD with the index SNP, if the index SNP is in LD with or located in a cardiac DHS, and if the locus contains cardiac or blood eQTLs

Abbreviations: *Chr* chromosome, *CA* coded allele, *CAF* coded allele frequency, *SE* standard error, *Non-red SNPs* non-redundant SNPs

**Table 2:** Description of novel PR loci among those of European descent

European ancestry index SNPs in novel PR loci									Non-red SNPs	AA PR	Missense	Cardiac DHS	Cardiac eQTL
Locus	SNP	Chr	Closest gene	CA	CAF	Beta (ms)	SE (ms)	*P*-value	*n*	*P* < 0.05	*r*^2^ > 0.8	*r*^2^ > 0.8	FDR < 0.05
**11**	rs4648819	1	*SKI*	G	0.11	−1.7	0.28	4.68E−10	1	–	–	–	–
**12**	rs7538988	1	*EPS15*	C	0.03	−2.1	0.37	1.14E−08	1	–	–	YES	–
**13**	rs12127701	1	*MYBPHL*	G	0.06	1.7	0.28	1.54E−09	1	–	*MYBPHL*	–	–
**14**	rs11264339	1	*KRTCAP2*	T	0.48	−0.7	0.11	5.94E−10	1	–	*EFNA1*	YES	–
**15**	rs397637	1	*OBSCN*	T	0.28	0.8	0.12	7.11E−10	1	–	*OBSCN*	YES	–
**16**	rs3856447	2	*ID2*	A	0.39	1.2	0.11	1.20E−26	2	–	–	YES	–
**17**	rs2732860	2	*TMEM182*	G	0.52	−0.9	0.11	3.03E−15	2	–	–	YES	*TMEM182*
**18**	rs13018106	2	*FIGN*	C	0.42	−0.8	0.12	1.53E−11	1	YES	–	YES	–
**19**	rs922984	2	*TTN*	T	0.07	1.5	0.23	1.79E−11	2	–	*TTN*	YES	–
**20**	rs9826413	3	*EOMES*	T	0.06	2	0.36	1.69E−08	1	–	–	–	–
**21**	rs900669	3	*FRMD4B*	A	0.25	0.8	0.13	5.71E−09	1	YES	–	YES	–
**22**	rs13087058	3	*PDZRN3*	C	0.37	−1	0.12	5.82E−17	1	–	–	YES	–
**23**	rs16858828	3	*PHLDB2*	C	0.18	0.9	0.15	2.41E−08	1	–	–	YES	–
**24**	rs6441111	3	*CCNL1*	C	0.52	0.8	0.13	6.96E−11	1	YES	–	YES	*LINC00881*
**25**	rs7638853	3	*SENP2*	A	0.34	−0.7	0.12	2.44E−08	1	–	*SENP2*	YES	–
**26**	rs17446418	4	*CAMK2D*	G	0.26	0.8	0.13	3.41E−09	1	–	–	YES	–
**27**	rs3733409	4	*FAT1*	T	0.13	0.9	0.17	2.67E−08	1	YES	*FAT1*	YES	*FAT1*
**28**	rs7729395	5	*PAM*	T	0.05	2.4	0.37	1.00E−10	1	–	*PAM*	–	–
**29**	rs11763856	7	*TBX20/HERPUD2*	T	0.03	3.1	0.49	4.47E−10	2	YES	–	YES	–
**30**	rs2129561	7	*MKLN1*	A	0.42	−1	0.12	3.39E−15	1	–	–	–	*LINC-PINT*
**31**	rs881301	8	*FGFR1*	C	0.41	0.8	0.12	5.04E−10	1	–	–	YES	–
**32**	rs12678719	8	*ZFPM2*	G	0.27	0.8	0.13	3.77E−10	1	–	–	–	–
**33**	rs12359272	10	*ALDH18A1/SORBS1*	A	0.37	1	0.13	3.68E−16	2	–	–	YES	–
**34**	rs12257568	10	*SH3PXD2A/OBFC1*	T	0.41	1	0.12	5.83E−18	2	YES	–	–	–
**35**	rs1372797	11	*NAV2*	T	0.12	−1.1	0.18	2.36E−09	2	–	–	YES	–
**36**	rs11067773	12	*MED13L*	C	0.09	−1.3	0.23	1.02E−08	1	–	–	–	–
**37**	rs718426	13	*EFHA1*	G	0.41	−1.2	0.11	3.25E−24	1	–	–	YES	–
**38**	rs2585897	13	*XPO4*	A	0.16	1.2	0.15	9.28E−16	1	YES	–	YES	–
**39**	rs9590974	13	*LRCH1*	C	0.34	1.1	0.12	1.02E−19	1	–	–	YES	–
**40**	rs11465506	14	*IL25/MYH6*	A	0.02	−6.4	1.04	7.06E−10	2	YES	*MYH6*	YES	–
**41**	rs4901308	14	*FERMT2*	T	0.19	−0.8	0.15	2.04E−08	1	–	–	–	–
**42**	rs17767398	14	*SNORD56B SIPA1L1*	G	0.26	1	0.13	6.44E−13	1	–	–	YES	–
**43**	rs904974	15	*TLE3*	T	0.16	1.1	0.19	4.53E−08	1	YES	–	–	–
**44**	rs1984481	17	*MYOCD*	C	0.54	−0.8	0.12	1.37E−11	1	–	–	YES	–

For each of the 34 novel loci, we list the number of non-redundant signals, whether this locus is nominally significant in African Americans, if missense SNPs are in LD with the index SNP, if the index SNP is in LD with or located in a cardiac DHS, and if the locus contains cardiac or blood eQTLs

*Chr* chromosome, *CA* coded allele, *CAF* coded allele frequency, *SE* standard error, *Non-red SNPs* non-redundant SNPs

### Putative functional variants

To assess the functional relevance of the identified SNPs, we used the 1000G reference panel to identify variants in high LD with the index SNPs. We then examined whether those variants were either nonsynonymous variants or fell within putative regulatory regions. Ten of the 44 loci had missense variants in high LD (*r*^2^ > 0.8) with the index SNP (Tables 1 and 2, Supplementary Data 4). *TTN*, in particular, was enriched for missense SNPs, with the top signal and approximately one-third of the 47 genome-wide significant TTN SNPs being missense (Fig. 2b). To examine the possible impact of these amino acid substitutions on protein structure or function, we used two prediction algorithms, Sift^9^ and PolyPhen-2^10^. The vast majority of the genome-wide significant missense variants at the 44 loci were categorized as tolerated by Sift and benign by PolyPhen-2, consistent with modest effects on PR interval not subjected to purifying selection (Supplementary Data 4).

Expression quantitative trait locus (eQTL) analysis using RNA-seq data suggests that index SNPs in nearly half of the identified loci (20/44) are associated with *cis* gene expression in cardiac left atrial appendage (LAA) tissue (*n* = 230) (Supplementary Data 5). Of these, we identified co-localizing variants that jointly affect both molecular expression and the PR phenotype to provide intuition regarding the candidate gene that may play a role in atrial conduction (Supplementary Data 6). Several points are worth highlighting. First, for most of the loci, the eQTL associations are for the gene nearest the index SNP, but for nearly one-quarter, they are not. Second, certain SNPs can be promiscuous in that they are associated with the transcript expression of multiple different genes (e.g., rs6599250 is associated with both *SCN5A* and *SCN10A* expression). Third, most of the eQTL associations found in cardiac atrial tissue—e.g., associations with *SCN10A, MEIS1*, *CAV1*, *FAT1*, and *TMEM182* transcripts—were not found in whole blood samples, even at nominal significance (Supplementary Data 6), despite larger sample sizes (*n* = 369 GTEx blood samples using RNA-seq and *n* = 5311 blood samples assayed by Illumina gene expression array). Similarly, while most cardiac eQTLs were identified in both atrial and ventricular tissue, *TMEM182* and *CAV1* eQTL associations are identified only in atrial tissue (Supplementary Data 6). Fourth, certain SNPs are associated with transcript expression levels of different genes, depending on the tissue being examined. For instance, rs2732860 was associated with *TMEM182* expression in atrial tissue but with *MFSD9* expression in blood, again suggesting tissue-specificity for SNP–eQTL associations. Taken together, these data underscore the importance of examining eQTL data in tissue types relevant to the trait of interest: even with a modest study size of 230 cardiac atrial samples, a notable number of eQTL associations were uncovered.

The majority of loci (30/44) contain index SNPs that lie in, or are in high LD with, regulatory regions of the genome that are marked by deoxyribonuclease I (DNAse I) hypersensitivity sites (DHSs), lending further support to the hypothesis that regulation of gene-expression plays an important role in determining PR interval (Tables 1 and 2). To provide insight into the cellular and tissue structure of the phenotype, we examined *P*-values of all SNPs in the PR meta-analysis and assessed cell- and tissue-selective enrichment patterns of progressively more strongly associated variants to explore localization of signal within specific lineages or cell types. As would be expected for the cardiac phenotype of PR interval, we found enrichment of signal in cardiac DHSs compared with DHSs from other tissue types (Supplementary Fig. 5). Interestingly, the second most enriched tissue DHSs were in those that regulate microvascular endothelial cells, complementing our findings (noted below) that there is an enrichment in genes involved in blood vessel morphogenesis. These findings possibly reflect the involvement of an overlapping set of transcription factors (e.g., *CAV1, NKX2-5, EFNA1, FGFR1, MEIS1, TBX5, WNT11, TBX20, ARHGAP24,* and *MYOCD)* influencing both cardiac and vascular development during mesodermal differentiation and development.

### Molecular function and biological processes of PR genes

Although extensive LD among common variants and the incompleteness of the HapMap reference panel preclude an unambiguous identification of the functional variant or the culprit gene, we used the following criteria to implicate genes in 37 of the 44 loci: (1) the gene selected was the only nearby gene (within a ±500 kb window); (2) the gene selected has a missense variant in high LD (*r*^2^ > 0.8) with the index SNP; or (3) the index SNP was associated with cardiac transcript expression levels of the selected gene (Tables 1 and 2). The set of implicated genes, detailed in Supplementary Note 3, showed strong enrichment for genes (permutation false discovery rate (FDR) *<* 1.0 × 10^−4^) involved in cardiac development, specifically the cardiac chambers and His-Purkinje system development (Supplementary Data 3). Other notable biological processes include the development of the vasculature and cardiac myocyte cell differentiation. The molecular function and cellular component of the identified genes were largely enriched for transcription factors, ion-channel related genes, and cell junction/cell signaling proteins (Supplementary Data 3).

### Clinical relevance of PR-associated loci

To examine the clinical relevance of the identified loci, we intersected the PR genes with gene membership from multiple knowledge bases encompassing over 4000 human diseases. The most highly over-represented conditions (permutation FDR < 1.0 × 10^−3^) are heart diseases including congenital abnormalities and heart failure, sick sinus syndrome and sinus arrhythmia (phenotypes related to the sinus node which houses the pacemaker cells that generate heartbeats), heart block (related to cardiac conduction between atria and ventricles), and AF (Supplementary Data 7).

**Fig. 3:**
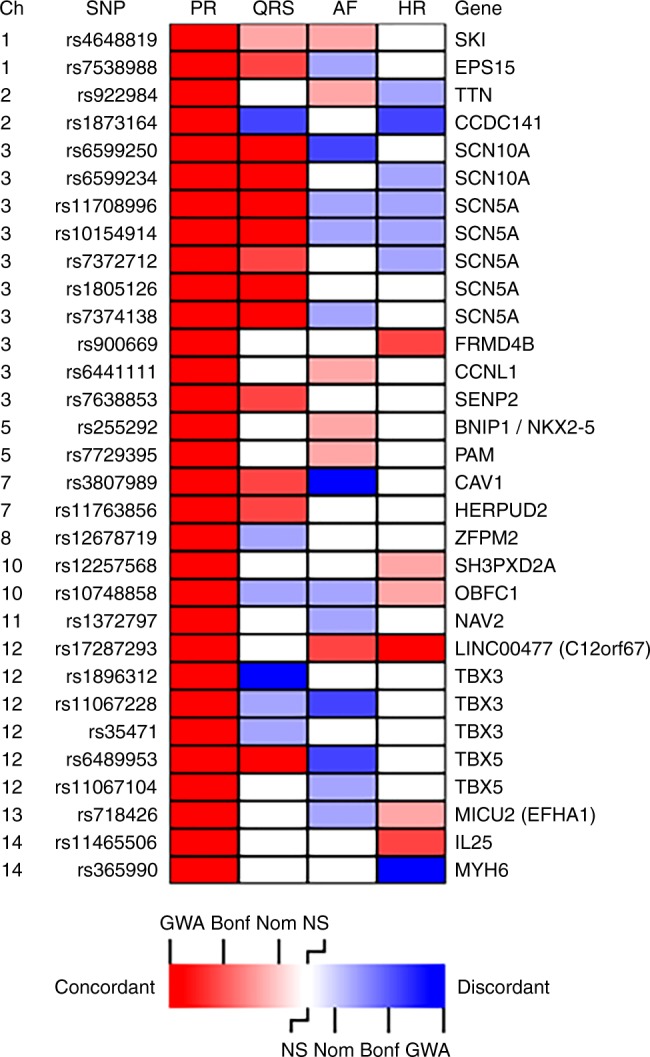
Heatmap showing overlapping loci between four traits. For each locus associated with PR interval, we tested the strength of the association and direction of effect for three related traits: QRS duration, atrial fibrillation, and heart rate. While the genetic bases of these three traits show a distinct overlap with that of PR interval, we observe for each trait overlapping loci with both concordant and discordant associations, with some variants that prolong PR interval prolonging QRS duration or increasing heart rate (concordant associations), whereas others shorten QRS duration or decrease heart rate. Similarly, some variants that prolong PR interval increase AF risk (concordant association) while others decrease AF risk (discordant).  Red color indicates concordant association with increasing PR associated with increasing QRS, or higher risk of AF, or higher HR. Blue color indicates discordant association of shortened QRS, or lower risk of AF, or lower HR.  Intensity of color indicates significance of association: GWA (GWAS significant association), Bonf (Bonferroni corrected significance), Nom (nominal significance), NS (not significant)

We examined the significant PR-associated SNPs for their effect on heart rate^11^, QRS interval (measure of ventricular conduction)^12^, and AF^13^. All 61 non-redundant SNPs from 44 independent loci were examined. Over half of the non-redundant SNPs (31/61) representing 20 loci were also associated with at least one of the other electrical phenotypes (Supplementary Data 8, Fig. 3). The cardiac sodium channel genes, *SCN5A* and *SCN10A*, clearly play a critical role in cardiac electrophysiology. PR prolonging variants in these genes are also associated with prolonged QRS duration, and some though not all variants at this locus are associated with lower heart rate, and lower risk of AF (Fig. 3). The role of transcription factors in cardiac electrophysiology is equally complex. Several T-box containing transcription factors, important for cardiac conduction system formation in the developing heart, are associated with PR interval. Although *TBX3* and *TBX5* sit close together on chromosome 12, the top PR prolonging allele in *TBX5* prolongs QRS and decreases AF risk while the top PR prolonging allele in *TBX3* shortens QRS duration while also decreasing AF risk. The PR prolonging variant near *TBX20* prolongs QRS duration but is not associated with AF risk (Fig. 3). Overall, eight of the 13 transcription factor genes associated with PR interval were also associated with at least one other atrial or atrioventricular electrical phenotype.

PR interval prolongation may reflect conduction disease, and prolonged PR interval is a risk factor for pacemaker implantation (~25% increase in the risk of pacemaker implantation for each 10 ms increase in PR duration above the median in the Copenhagen Study)^14^. We examined whether PR prolonging variants were associated with higher risk of pacemaker implantation among ~370,000 individuals from the UKBiobank, of whom 1074 require pacemaker implantation. Using Mendelian randomization, we show that while prolongation of PR interval is causally related to pacemaker implantation, the MR estimate of the causal effect is smaller (OR = 1.14/10 ms increase in PR interval) than the effect size seen observationally for PR on pacemaker implantation, suggesting that acquired factors such as heart disease may also play an important role (Supplementary Fig. 6).

### PR and QRS intervals

Many loci regulate both atrial/atrioventricular (PR interval) and ventricular (QRS) depolarization and conduction: 12 of the 44 PR loci were nominally associated with QRS duration (Supplementary Data 7) and, similarly, 17 of 22 previously identified QRS loci were at least nominally associated with PR interval (Supplementary Data 9). Several intriguing findings are worth highlighting. First, while SNPs in most loci that are associated with prolonged PR are also associated with prolonged QRS, two loci have genome-wide significant discordant PR–QRS relationships, in which prolonged PR variants are associated with shorter QRS duration (*TBX3* and *SNORD56B)*; Supplementary Data 8, Fig. 3, Supplementary Fig. 7b. Second, although *TBX20* plays a crucial role in the development of the cardiac conduction system, the SNPs that are associated with atrial and atrioventricular conduction (PR) differ from those related to ventricular conduction (QRS) (index SNP PR rs11763856, index SNP QRS rs1419856, *r*^2^ = 0.001). A better understanding of the influence of these specific regions on cardiac conduction will require further investigation.

### PR interval and atrial fibrillation

One-third (18/61) of PR index SNPs were nominally associated with AF. For six of these SNPs, the alleles associated with prolonged PR are associated with increased AF risk, whereas for 12, the alleles associated with prolonged PR are paradoxically associated with lower AF risk. This observation is consistent with the relationship between PR interval and AF described in population studies, where we showed that while both short (<120 ms) and long (>200 ms) PR intervals are associated with increased AF risk, short PR interval is associated with higher risk than long PR interval^13^. For both concordant (meaning relationships where the PR prolonging variant is associated with increased AF risk) and discordant PR–AF relationships, the larger the SNP effect size for PR interval, the larger the odds ratio for association with AF (Supplementary Fig. 7a). The *CAV1* index SNP associated with increased PR interval and decreased AF risk reached genome-wide significance for both phenotypes. Furthermore, of 23 previously described AF GWAS loci, 11 were at least nominally associated with PR interval^15^. Interestingly, despite adequate power to identify modest associations, several loci, including *PITX2*, the most significant AF GWAS locus, showed no association with PR interval, though a prior report found modest nominal association with P-wave duration (Supplementary Data 9)^16^. Therefore, these loci may have a mode of action independent of atrial and atrioventricular depolarization or conduction.

### PR interval and heart rate

Ten PR loci were nominally associated with heart rate, including two sarcomeric proteins, *MYH6* and *TTN*. At the *MYH6* locus, a known heart rate locus^4^, variant rs365990 is associated with prolonged PR interval and with slower heart rates, whereas a non-redundant second *MYH6* signal (<20 kb away; rs11465506), the allele associated with prolonged PR is associated with faster heart rates. We then examined heart rate SNPs for association with PR and found half of the heart rate SNPs were associated with PR interval, with both concordant and discordant effects. Adjusting for heart rate in the regression model did not impact the effect size or significance of the PR–genotype associations, even though resting heart rate is modestly associated with PR interval (Supplementary Fig. 8).

### Cross-trait genome-wide meta-analyses

Finally, we performed joint phenotype analyses, with PR–heart rate, PR–QRS, and PR–AF as outcomes, to increase the power of finding loci involved in cardiac electrical activity. As described above, prolonged PR variants can have either a concordant or discordant association with another electrical phenotype. Therefore, we modeled the outcome for each joint analysis in two ways: with a variant having a concordant effect on PR–QRS, PR–HR, and PR–AF, and a discordant effect (Supplementary Figures 6a–c). These analyses yielded three novel loci associated with atrial electrical activity: one related to atrial and ventricular conduction (from PR–QRS analyses) and two related to atrial electrical activity and arrhythmias (from PR–AF analyses); (Supplementary Tables 1 and 2, Supplementary Note 3, Supplementary Fig. 9). Additional support for association of these loci were obtained by an analysis limited to cardiac DHS sites, and by trans-ethnic meta-analysis with African Americans, described below, lending further support to the validity of these associations (Supplementary Table 1, Supplementary Fig. 9).

### Trans-ethnic analyses

Our study had less power to find associations among African Americans (*n* = 13,415) than among European-descent individuals (*n* = 92,340). Nonetheless, 16 of the 44 European-identified loci nominally replicated among African Americans, suggesting that a large proportion of genetic associations with PR interval are shared between the two ethnic groups (Supplementary Data 9). For European-descent GWAS PR SNPs at least nominally associated with PR among African Americans, the estimated effect was in the same direction for the two population (Supplementary Fig. 7d).

Examining only the index signal may underestimate the true number of locus associations that replicate. Differences in LD structure between the genomes of individuals of European descent and those of African American descent cause dissimilar patterns of SNPs associated with PR interval. For instance, the *TBX5* locus index SNP rs6489953 is part of a large LD block associated with PR interval among individuals of European descent. This SNP is not significantly associated with PR interval among African Americans (beta = 0.04, *P* = 0.90, Supplementary Data 8, Fig. 2c). There is, however, a strong SNP–PR association signal in the *TBX5* among African Americans (index SNP rs7955405, beta = 1.16, *P* = 9.2 × 10^−16^ in African Americans), Fig. 2c. This SNP is in high LD with rs6489953 among European descent individuals (HapMap CEU *r*^2^ = 0.62), but not among the population from African descent (HapMap YRI *r*^2^ = 0.03). Hence, examination of only the top European descent index signal would miss the association among African Americans. Furthermore, interrogation of the *TBX5* locus among African Americans narrows the association block, allowing for fine mapping of the association signal (Fig. 2c). A second noteworthy interethnic difference is that there are SNP associations among those of European descent, for instance, rs1896312 in *TBX3*, where despite adequate power, no association could be established among African Americans (Fig. 2c).

Our trans-ethnic GWAS meta-analysis of PR interval among 13,415 African Americans and 92,340 European-ancestry individuals identified five additional novel loci associated with atrial and atrioventricular conduction (PR interval) (Supplementary Table 1, Supplementary Fig. 9).

## Discussion

Our GWAS meta-analytic study of over 92,000 individuals of European ancestry identified 44 loci associated with cardiac atrial and atrioventricular conduction (PR interval). The implicated genes at these loci show strong enrichment for genes involved in processes related to cardiac conduction, namely, cardiovascular system development and, specifically, in development of the cardiac chamber and bundle of His. Similarly, diseases overrepresented by these genes are processes related to arrhythmias and heart block, consistent with the current knowledge of the physiology and epidemiology of cardiac atrial conduction.

Using HapMap^17^ imputation, we tested over 2.7 million SNPs, and while we did not directly test all common variants with this approach, nor did we test low-frequency variants (with minor allele frequencies below 1%), we identified many index SNPs in LD with functional variants, either through amino acid changes or involvement in gene regulation. For most newly identified loci, we are able to pinpoint a gene that potentially may be causative, either because the index SNP (or a SNP in high LD with it) is a missense variant in the gene, or because it regulates the expression of the gene. Regulation of gene expression can be tissue-specific, and our findings underscore the importance of examining eQTL data in tissue types relevant to the trait of interest.

A total of 34 novel loci were identified for PR interval in Europeans. Several have been identified previously in a related phenotype or in a different ancestral population, complementing our findings. Two loci, *EFHA1* and *LRCH1*, were previously identified for association with the PR segment^18^. In addition, the novel locus *CAMK2D* was found to be associated with P-wave duration, and *MYH6* with P-wave duration and P-wave terminal force^19^. The *ID2* locus on chromosome 2 was found in a GWAS on PR interval in Hispanic/Latino population^20^. A locus that was identified in two studies in Asian population^21, 22^, *SLC8A1*, did not reach genome-wide significance in our meta-analysis, but was suggestive with the strongest SNP being rs13026826 (beta for A-allele: 0.278, *P* = 1.036 × 10^−6^).

Contrasting meta-analyzed association results from European descent individuals with results from a smaller sample of African Americans, we find that, with few exceptions, a large proportion of genetic associations with PR interval are shared between the two ethnic groups. We then combined data from Europeans and African Americans in a trans-ethnic meta-analysis, allowing us to find additional loci. With over 105,000 samples in total, our power was ~80% to find a significant association at common variants that explain ~0.04% of the variance in PR interval. Future studies should examine sequence or other data that provide better assessment of rare and common functional variants, as was done previously for *SCN5A*^7^.

We also combined our results on PR interval with previously published results on heart rate, QRS duration, and AF, and identified loci contributing to atrial arrhythmias and cardiac conduction. We observed significant pleiotropy of effect of these SNPs, with over half of the SNPs associated with PR interval (atrial conduction) in the study also associated with ventricular conduction (QRS interval), atrial arrhythmias (AF), and heart rate (RR interval).

Our series of GWAS, including transethnic and cross-trait meta-analytic studies, has identified 50 loci, 40 of which are novel, associated with cardiac atrial and atrioventricular electrical activity among individuals of European and African ancestry. Understanding the biology of a trait in this way provides insight into related disease processes and may help identify potential approaches to drug therapy.

## Methods

### Meta-analysis of PR interval

We included 32 cohorts comprising 92,340 individuals. Ethical review boards of the respective cohorts approved the studies and informed consent was obtained from all subjects. Detailed information on the participating cohorts is in Supplementary Note 1. Each cohort conducted a GWAS on PR-interval measured on baseline EKG recordings of healthy individuals. Subjects were excluded from analysis based on a set of criteria employed by all participating cohorts, including presence of AF on the baseline EKG, a history of myocardial infarction or heart failure, extreme PR values (≤80 ms or ≥ 320 ms), Wolff–Parkinson–White syndrome, pacemaker implantation, the use of class I and III blocking medications (ATC code prefix C01B) or digoxin, and pregnancy. Age, sex, height, body mass index, and principal components were included as covariates, as well as study site if applicable. We did not exclude or correct for beta blocker and/or calcium channel blocker use. As a sensitivity analysis, we further adjusted for these variables in the largest cohort, ARIC. No appreciable change was observed in the effect estimates (*r* > 0.99). Analyses were stratified by ethnicity to maintain a homogeneous population with similar LD patterns across cohorts. Low-quality SNPs were removed based on stringent quality control criteria and untyped SNPs were imputed using HapMap 2 as reference panel prior to the association analysis.

Summary level data from all cohorts were collected and stringent quality control was applied to the data, removing SNPs with extreme beta and/or standard error values, or with poor imputation quality. Per cohort, SNPs with an imputation quality below 0.1 or above 1.1 were removed, as well as SNPs with a beta or standard error greater than 1000 or less than −1000. Next, SNPs were removed based on manual examination of quantile–quantile plots stratified for minor allele frequency and for imputation quality; SNPs in strata with early departure from the null were excluded for that specific cohort. Remaining SNPs were meta-analyzed using an inverse-variance fixed effects model^23^, correcting for per cohort inflation factors (lambda). Two cohorts, AGES and deCODE, contained a small percentage of overlapping samples: approximately 5% based on projections as well as based on *Z*-statistics from each study using the program METAL (https://genome.sph.umich.edu/w/images/7/7b/METAL_sample_overlap_method_2017-11-15.pdf). To account for this overlap and to adjust for a corresponding inflation of the test statistic, we separately meta-analyzed AGES and deCODE, corrected for the corresponding genomic inflation factor (1.089), and included all corrected association results into the overall meta-analysis.

We conducted the meta-analysis by using three independent analysts and two different software packages: MANTEL (http://debakker.med.harvard.edu/resources.html) and METAL (http://www.sph.umich.edu/csg/abecasis/metal/). All results were extremely concordant, reflecting a robust analysis. In total, 2,712,613 SNPs were tested for association with PR interval. Results were considered statistically significant at a *P* *=* 5 × 10^−8^, a figure that reflects the estimated testing burden of one million independent SNPs in samples of European ancestry^24^. Regions harboring association signals were visualized using SNAP^25^.

### GWiS

To identify non-redundant association signals within each locus and calculate the variance explained, we implemented the GWiS method, which aggregates the statistical support for multiple independent effects at a locus using a reference LD matrix^6^. A locus is defined as the genomic region flanked by the 5′ and 3′ most genome-wide significant signal, plus 100 KB of flanking sequence on each end. For each locus, GWiS uses Bayesian model selection to find the number of independent effects and the SNPs that best tag them, choosing the SNPs that maximize the posterior probability in a greedy search. In each step, the SNP that gives the greatest increase in the posterior probability is added into the model, and this step is repeated until no more SNPs increase the posterior probability.

The SNPs selected by the Bayesian model selection are then used in a multivariate linear regression to calculate the variance explained. We modified the original implementation of GWiS to use the meta-analysis results as input^26^.

GWiS was applied to the GWAS meta-analysis, making use of pairwise SNP *r*^2^ estimates from the ARIC study. GWiS estimated 61 non-redundant signals of association at the 40 genome-wide significant loci (Supplementary Data 4).

### Gene selection, gene enrichment, clinical relevance

Although extensive LD among common variants and the incompleteness of the HapMap reference panel preclude an unambiguous identification of the functional variant or the culprit gene, we used the following criteria to implicate genes in 37 of the 44 loci: (1) the gene selected was the only nearby gene (within a ±500 kb window); (2) the gene selected has a missense variant in high LD (*r*^2^ > 0.8) with the index SNP; or (3) the index SNP was associated with cardiac transcript expression levels of the selected gene.

We performed over-representation enrichment analysis on PR genes relative to the entire human genome by leveraging several human disease knowledge bases including PharmGKB (~3500 disorders, www.pharmgkb.org), Human Phenotype Ontology (~4000 common diseases, http://human-phenotype-ontology.github.io/), and DisGeNET (http://www.disgenet.org). The analysis used the program WebGestalt (www.webgestalt.org), which computed over-representation *P*-values based on hypergeometric distributions^27^. To further increase our confidence in gene set analysis, we also applied gene set enrichment analysis (GSEA) to the entire GWAS gene list rank ordered based on their association *P*-values. We used the latest GO dataset available at the Molecular Signatures Database (v6.1). We performed 1000 random permutations and used an FDR < 0.01 threshold to identify enriched GO categories. The highly enriched GO annotations identified using the parametric approach were also significant based on the permutation method, and we report only GO categories that were significantly enriched at FDR < 0.01 common to both the parametric and nonparametric procedures.

Multiple hypothesis testing was addressed using Benjamini–Hochberg’s FDR adjustment of the enrichment *P*-values, and an FDR threshold < 0.01 was used to designate significantly over-represented disease states. We applied the same approach using WebGestalt to identify enriched functional categories (FDR < 0.01) based on Gene Ontology annotations of molecular function, cellular component, and biologic process^28^.

### Functional variants in significant loci

We annotated the 61 index SNPs and nearby SNPs in LD with the index SNPs (within 1 Mb and with *r*^2^ > 0.8 in 1000 Genomes Phase 1 CEU) and tested all non-synonymous SNPs with PolyPhen-2^10^ and SIFT^9^ to predict the functional implication.

We performed an eQTL analysis using the 61 PR index SNPs. We examined eQTL associations from LAA and validated findings in tissue from right atrial appendage (RAA). For comparison, we also evaluated left ventricular tissue as well as peripheral whole blood.

Human LAA tissue was obtained with consent from 223 European-American patients undergoing cardiac surgery for treatment of AF, valvular, and/or coronary artery disease. Use of discarded surgical tissue was approved by the Institutional Review Board of the Cleveland Clinic. Before 2008, verbal consent was obtained and documented in the medical records in a process approved by the Cleveland Clinic Institutional Review Board. From 2008 onward, patients provided separate Institutional Review Board-approved written informed consent. AF history, type of AF, and other clinical data were collected in a research database. LAA tissue was also obtained from 12 non-failing donor hearts not used for transplant with written consent for research use provided by the family. Donor information included age, race, and sex. The Cleveland Clinic Institutional Review Board approved the tissue studies included in this report. Demographic characteristics of the study population have been summarized Table 1, “subjects of European descent” column^29^.

LAA specimens were snap frozen in liquid nitrogen and stored at −80 °C. Total RNA was extracted 50–100 mg tissue using the Trizol protocol. Tissue was homogenized with sterile Omni Tip Disposable Generator Probes (Omni International). RNA processing and sequencing and DNA genotyping have been described^29^. Library generation for RNAseq was done at the University of Chicago Genomics Facility using standard Illumina protocols. Samples were filtered based on RNA quality. Unstranded 100-bp paired-end sequencing was performed on the Illumina HiSeq 2000 platform and multiplexed to six samples across two lanes. Samples were demultiplexed and aligned to hg19 using TopHat (v2.0.4)11 with the default options. Reads from exactly matched PCR duplicates were marked using Picard tools (https://broadinstitute.github.io/picard/) and excluded from further analysis. Sequence reads were mapped to the human genome to derive a digital count of the expression of genes, which were defined using the Ensembl (version 71) gene catalog.

DNA was extracted from 25–50 mg homogenized LAA tissue (as above) using the DNAzol protocol. DNA was genotyped using Illumina Hap550v3 and Hap610-quad SNP microarrays. SNP data were imputed to 1000 Genomes Project phase 2 yielding ≈19 million SNPs, using IMPUTE10 after filtering out variants falling below 0.5 on IMPUTE’s information statistic. For the eQTL analysis, we excluded SNPs with <5% minor allele frequency, resulting in roughly 6.8 million SNPs.

Methods for LAA eQTL analysis have been described^29^. Expression counts were obtained from aligned files using htseq counts against the human Ensembl gene annotation. On average, 26 million paired-end read fragments aligned to this annotation of the transcriptome across all of our samples. Reads were quantile-normalized, and gene counts for eQTL analysis were variance-stabilized transformed using the R package Deseq2. Expression of each gene was adjusted by the following covariates: sex, genetic substructure based on four multidimensional scaling factors, and 25 expression surrogate variable analysis (SVA) covariates. The SVA method is similar to principal component analysis, which uses unsupervised mathematical models to separate out the high variance components in high dimensional data. Thus, without manual normalization, the SVA method corrects for potential large effectors of gene expression such as read-depth, batch effects, and other technical variables, as well as environmental and disease effects such as AF status, history of structural heart disease, coronary artery disease, etc. Surrogate variables were calculated from the variance-stabilizing transformation data using the sva package. eQTL analyses were performed using MatrixeQTL (2.1.0) to test associations between genotype and variance-stabilizing transformation counts. *β*-coefficients were calculated as the additive effect of 1 allelic difference on log_2_ gene expression. The QVALUE package was used to estimate FDR from the complete list of cis-eQTL SNP/genome-wide expressed gene pairs *P* values. Linear regression and Q–Q plot comparison of the LAA eQTLs with selected tissues were performed using the version 6p analysis of GTEx project.

We also performed eQTL associations in 5311 samples from peripheral blood. Those methods have also been described previously^30^. In brief, Illumina Gene expression data for each dataset was obtained and sequences mapped against the human genome build 36 (Ensembl build 54, Hg18). Highly stringent alignment criteria were used to ensure that probes map unequivocally to one single genomic position. Genotype data was acquired using different genotyping platforms, and harmonized by imputation (HapMap2 CEU).

Gene expression data was quantile-normalized to the median distribution, and subsequently log_2_ transformed. The probe and sample means were centered to zero. Gene expression data was then corrected for possible population structure by removal of four multi-dimensional scaling components using linear regression.

After normalization of the data, we performed *cis*-eQTL mapping. eQTLs were deemed *cis*-eQTLs when the distance between the SNP chromosomal position and the probe midpoint was less than 250 kilobases (kb). eQTLs were mapped using a Spearman’s rank correlation on the imputed genotype dosage values. We used a weighted Z-method for subsequent meta-analysis. We permuted the sample identifiers labels of the expression data and repeated this analysis ten times. In each permutation, the sample labels were permuted. We then corrected for multiple testing by controlling the FDR at 0.05, by testing each *P*-value in the real data against a null-distribution created from the permuted datasets.

Significant associations from LAA eQTL analyses were replicated using pre-calculated eQTLs from GTEx. We accessed full single tissue cis-eQTL analyses for left ventricle, RAA, and whole blood from GTEx v7, accessed on 22nd March, 2018. Samples with genotype and expression data for eQTL analyses were *n* = 272, *n* = 264, and *n* = 369, for left ventricle, RAA, and whole blood, respectively.

For each of the 61 nonredundant SNPs in the 44 independent loci, we identified the probes/genes for which there was a *cis-*eQTL association. We also identified the most significant SNP (eSNP) associated with that gene. Frequently, the eSNP and our SNP of interest were in high or near perfect LD and represented the same signal (see Supplementary Figures 2 and 4 for example of *MEIS1* demonstrating strong co-localization).

Our aim for identifying co-localizing genetic variants that jointly affect both molecular expression and the PR phenotype is to provide intuition regarding the candidate gene that may play a role in atrioventricular conduction. In any given locus, we identify a candidate gene from eQTL data if it meets the following three criteria: (1) the SNP–transcript association in LAA is significant at a threshold of genome-wide *q* < 0.05; (2) there is evidence of co-localization in that the PR-GWAS index SNP and the top LAA SNP are in high LD (>0.90) OR there is evidence that the association remains significant in conditional analysis examining the PR-GWAS index SNP adjusted for the top LAA SNP (*P* < 0.01); and (3) the findings from the LAA replicate (*P* < 0.05) in GTEx data from the RAA. It is important to note that our replication tissue from GTEx is RAA whereas our discovery tissue is LAA. While we only claim as candidate genes those that replicate, differences in eQTL associations between LAA and RAA are nonetheless interesting and noted in Supplementary Data 6.

At successively more stringent *P*-value thresholds, SNPs were evaluated for enrichment in tissue-specific DNAse I hypersensitive sites. SNPs from each PR association *P*-value bin were intersected with the complete set of DHS false discovery 5% hotspot regions identified in any of the 349 tissue or cell line samples available from Maurano et al.^31^. Intersections between GWAS SNPs and DHS regions were computed using the BEDOPS^32^ software. Fold enrichment was calculated by comparing the proportion of SNPs within each *P*-value bin to the background rate of all GWAS variants falling within the DHS sites for each tissue separately.

### Transethnic analyses

To search for additional loci involved in PR interval, results of a published GWAS on PR interval in African Americans^33^ were combined with our GWAS meta-analysis results in Europeans using inverse variance weighted fixed-effect models, correcting for the inflation factor of both cohorts. New loci were called if they reached statistical significance at a *P* ≤ 5 × 10^−8^, and if this locus was not significantly associated with PR interval in Europeans or African Americans separately (i.e., if none of the SNPs within one Megabase of the tested SNP reached *P* ≤ 5 × 10^−8^ in any of the population). SNP look-ups of index SNPs in Europeans were performed in African American GWAS results, to test for overlapping signals in both ethnicities that were not observed in African Americans because of the relatively low sample size (*n* = 13,415).

### Cross-trait meta-analyses

For the joint analysis of PR and AF, beta estimates and standard errors were used to generate *z*-scores (beta/se), which were then combined as (*z*_PR_ + *z*_AF_)/sqrt(2) to identify genetic variants that both increase PR interval and risk for AF, and as (*z*_PR_−*z*_AF_)/sqrt(2) to identify genetic variants that increase PR interval, but decrease the risk for AF. Genome-wide significance was set at 8.3 × 10^−9^, to account for the six tests performed across the three traits that were meta-analyzed with PR interval. Only loci that did not contain variants genome-wide significant separately for PR or AF were concerned novel.

To search for additional loci involved in atrial and ventricular cardiac conduction, we meta-analyzed our PR interval GWAS results with previously published QRS duration^8^ and RR interval^11^ results, respectively. We used sample size (*z*-score) weighted models[34] to identify variants that increase both PR interval and the second trait tested (either QRS duration or RR interval) and variants that increase PR interval but decrease risk for the second trait.

Genomic inflation factor lambda was 1.02 for concordant PR–QRS, 0.98 for discordant PR–QRS, 1.01 for concordant PR–RR, and 0.98 for discordant PR–RR. Therefore, we did not correct for these lambdas, even though the meta-analyses contain overlapping samples.

New loci were called if they reached statistical significance at a *P* ≤ 8.3 × 10^−9^, and if this locus was not significantly associated with PR interval nor with the second trait tested. SNP look-ups of index SNPs in PR interval were performed in QRS duration and RR interval, and also the other way around (QRS duration and RR interval index SNPs in PR interval) to test for overlapping signals.

### Association with pacemaker implantation

Association with pacemaker implantation was determined in the UKBiobank data for the 61 SNPs independently associated with PR interval. Samples were limited to unrelated whites of British ancestry (~370,000 samples), of whom 1074 had a pacemaker implanted. A logistic regression model was run, including covariates for age, sex, and 40 PCs to account for potential population substructure or other potential confounding. Inverse variance weights (IVW) Mendelian randomization was performed using the “MendelianRandomization” package in R. Results were consistent with those produced by MR-EGGER and MR-Median regression.

### Data availability

The full meta-analysis results are available for download through the CHARGE repository in dbGaP: http://www.chargeconsortium.com/main/results

## Electronic supplementary material

Supplementary Information

Description of Additional Supplementary Files

Supplementary Data 1

Supplementary Data 2

Supplementary Data 3

Supplementary Data 4

Supplementary Data 5

Supplementary Data 6

Supplementary Data 7

Supplementary Data 8

Supplementary Data 9

## References

[1] Kannel WB, Benjamin EJ (2009). Current perceptions of the epidemiology of atrial fibrillation. Cardiol. Clin..

[2] Hanson B (1989). Genetic factors in the electrocardiogram and heart rate of twins reared apart and together. Am. J. Cardiol..

[3] Pfeufer A (2010). Genome-wide association study of PR interval. Nat. Genet..

[4] Holm H (2010). Several common variants modulate heart rate, PR interval and QRS duration. Nat. Genet..

[5] Bulik-Sullivan BK (2015). LD Score regression distinguishes confounding from polygenicity in genome-wide association studies. Nat. Genet..

[6] Huang H, Chanda P, Alonso A, Bader JS, Arking DE (2011). Gene-based tests of association. PLoS Genet..

[7] Magnani JW (2014). Sequencing of SCN5A identifies rare and common variants associated with cardiac conduction: Cohorts for Heart and Aging Research in Genomic Epidemiology (CHARGE) Consortium. Circ. Cardiovasc. Genet..

[8] Sotoodehnia N (2010). Common variants in 22 loci are associated with QRS duration and cardiac ventricular conduction. Nat. Genet..

[9] Kumar P, Henikoff S, Ng PC (2009). Predicting the effects of coding non-synonymous variants on protein function using the SIFT algorithm. Nat. Protoc..

[10] Adzhubei IA (2010). A method and server for predicting damaging missense mutations. Nat. Methods.

[11] Eijgelsheim M (2010). Genome-wide association analysis identifies multiple loci related to resting heart rate. Hum. Mol. Genet..

[12] van der Harst P (2016). 52 Genetic loci influencing myocardial mass. J. Am. Coll. Cardiol..

[13] Ellinor PT (2012). Meta-analysis identifies six new susceptibility loci for atrial fibrillation. Nat. Genet..

[14] Rasmussen PV (2017). Electrocardiographic PR interval duration and cardiovascular risk: results from the Copenhagen ECG study. Can. J. Cardiol..

[15] Christophersen IE (2017). Large-scale analyses of common and rare variants identify 12 new loci associated with atrial fibrillation. Nat. Genet..

[16] Thorolfsdottir RB (2017). A missense variant in PLEC increases risk of atrial fibrillation. J. Am. Coll. Cardiol..

[17] Frazer KA (2007). A second generation human haplotype map of over 3.1 million SNPs. Nature.

[18] Verweij N (2014). Genetic determinants of P wave duration and PR segment. Circ. Cardiovasc. Genet..

[19] Christophersen, I. E. et al. Fifteen genetic loci associated with the electrocardiographic P wave. *Circ. Cardiovasc. Genet*. **10**, e001667 (2017) 10.1161/CIRCGENETICS.116.001667. 10.1161/CIRCGENETICS.116.001667 PMC5567993 28794112

[20] Seyerle, A. A. et al. Genome-wide association study of PR interval in Hispanics/Latinos identifies novel locus at ID2. *Heart* **104**, 904–911 (2017). 10.1136/heartjnl-2017-312045 PMC6946379 29127183

[21] Hong KW (2014). Identification of three novel genetic variations associated with electrocardiographic traits (QRS duration and PR interval) in East Asians. Hum. Mol. Genet..

[22] Sano M (2014). Genome-wide association study of electrocardiographic parameters identifies a new association for PR interval and confirms previously reported associations. Hum. Mol. Genet..

[23] de Bakker PI (2008). Practical aspects of imputation-driven meta-analysis of genome-wide association studies. Hum. Mol. Genet..

[24] Pe’er I, Yelensky R, Altshuler D, Daly MJ (2008). Estimation of the multiple testing burden for genomewide association studies of nearly all common variants. Genet. Epidemiol..

[25] Johnson AD (2008). SNAP: a web-based tool for identification and annotation of proxy SNPs using HapMap. Bioinformatics.

[26] Chanda P, Huang H, Arking DE, Bader JS (2013). Fast association tests for genes with FAST. PLoS One.

[27] Wang, J., Vasaikar, S., Shi, Z., Greer, M. & Zhang, B. WebGestalt 2017: a more comprehensive, powerful, flexible and interactive gene set enrichment analysis toolkit. *Nucleic Acids Res.* **45**, W130-W137 (2017). 10.1093/nar/gkx356 PMC5570149 28472511

[28] The Gene Ontology Consortium (2017). Expansion of the Gene Ontology knowledgebase and resources. Nucleic Acids Res..

[29] Hsu J (2018). Genetic control of left atrial gene expression yields insights into the genetic susceptibility for atrial fibrillation. Circ. Genom. Precis. Med..

[30] Westra HJ (2013). Systematic identification of trans eQTLs as putative drivers of known disease associations. Nat. Genet..

[31] Maurano MT (2012). Systematic localization of common disease-associated variation in regulatory DNA. Science.

[32] Neph S (2012). BEDOPS: high-performance genomic feature operations. Bioinformatics.

[33] Butler AM (2012). Novel loci associated with PR interval in a genome-wide association study of 10 African American cohorts. Circ. Cardiovasc. Genet..

[34] Eijgelsheim, M. *Genetic Determinants of Heart Rhythm and Conduction Disorders*. PhD thesis, Erasmus University Rotterdam (2011).

[35] den Hoed, M. et al. Identification of heart rate-associated loci and their effects on cardiac conduction and rhythm disorders. *Nat. Genet.* **45**, 621–631 (2013). 10.1038/ng.2610 PMC3696959 23583979

