# Supplementary material for: PR interval genome-wide association meta-analysis identifies 50 loci associated with atrial and atrioventricular electrical activity
Source: Nat Commun. 2018 Jul 25;9:2904. doi: 10.1038/s41467-018-04766-9 (PMC6060178; doi:10.1038/s41467-018-04766-9)
Supplement: Supplementary file 1 — Supplementary Information [file 41467_2018_4766_MOESM1_ESM.docx]

**PR interval genome-wide association meta-analysis identifies 50 loci associated with atrial and atrioventricular electrical activity**

van Setten et al.

**Supplementary Notes**

**Supplementary Note 1: Study population and cohort information**

**European ancestry**

*AGES-Reykjavik*

The Reykjavik Study^1^ cohort originally comprised a random sample of 30,795 men and women born in 1907–1935 and living in Reykjavik in 1967. 2 A total of 19381 attended, resulting in 71% recruitment rate. The study sample was divided into six groups by birth year and birth date within month. One group was designated for longitudinal follow-up and was examined in all stages. One group was designated a control group and was not included in examinations until 1991. Other groups were invited to participate in specific stages of the study. Between 2002 and 2006, the AGES-Reykjavik study re-examined 5764 survivors of the original cohort who had participated before in the Reykjavik Study. PR interval duration was automatically measured from 12-lead electrocardiograms using the Marquette 12 SL analysis program.

*AMISH*

Participants were recruited from the Amish community of Lancaster County, PA.^2^ Examinations were conducted at the Amish Research Clinic in Strasburg, PA. The Institutional Review Board at the University of Maryland approved all protocols and informed consent was obtained, including permission to use their DNA for genetic studies. Study participants were enrolled within the 2000-2008 time period. 12-lead electrocardiograms (ECG) were acquired using standard methods with a GE Marquette digital recording system. The PR measurements were calculated by the Marquette computer system algorithm.

*ARIC*

The Atherosclerosis Risk in Communities (ARIC) Study^3^ is a prospective community-based study of cardiovascular disease and its risk factors. At baseline (1987-89), 15,792 men and women age 45-64 were recruited from 4 communities in the US (Washington County, Maryland; Forsyth County, North Carolina; Jackson, Mississippi; Minneapolis suburbs, Minnesota). Participants were mostly white in the Minnesota and Washington County field centers, white and African American in Forsyth County, and exclusively African American in the Jackson field center. ECGs were recorded on MAC PC Personal Cardiographs (Marquette Electronics Inc., Milwaukee, WI) and were subsequently submitted to a central reading center at the EPICORE Center (University of Alberta, Edmonton, Alberta, Canada) and thereafter to the Epidemiological Cardiology Research Center (EPICARE), Wake Forest University, Winston-Salem, NC. All ECGs were visually inspected for quality and legibility at their acquisition and by the reading centers and then stored in a digital format. The PR interval was determined as the mean duration in milliseconds from the P wave onset until the initiation of the QRS segment in the 12 ECG leads.

*BioVU*

BioVU^4^ is the Vanderbilt University Medical Center (VUMC) biorepository linked to de-identified electronic health records. The rationale, infrastructure, and oversight for BioVU have been previously described.^5^ In brief, the samples used in this study were obtained when BioVU followed an opt-out model; since January 2015, samples are collected under an opt-in model. DNA is extracted from blood collected as part of routine clinical care from VUMC outpatient clinics. The DNA samples were linked to a de-identified version of the patient’s EHRs, as defined by the Health Insurance Portability and Accountability Act (HIPPA) and are in accordance with provisions of Title 45, Code of Federal Regulations, part 46 (45 CFR 46) that define criteria for “non-human subjects” research.^6^ Electrocardiogram data were extracted from the EHR as previously described.^7,8^ Electronic phenotyping for this study included only normal ECGs and absence of any cardiac disease or abnormal electrolyte values. The electronic phenotyping strategy was developed and refined at VUMC as part of the larger electronic MEdical Records & GEnomics (eMERGE) network, an NHGRI- funded collaboration of five biobanks linked to EHRs in the United States.^9^ The algorithm was validated to a positive predictive value of 97%, and details are available on PheKB (<http://phekb.org/>). Deploying the algorithm at VUMC identified 2,334 European Americans (991 males and 1,343 females). BioVU DNA samples were genotyped at the Center for Genotyping and Analysis at the Broad Institute and the Center for Inherited Disease Research (CIDR) at Johns Hopkins University, and cleaned using the quality control pipeline developed by the eMERGE Genomics Working Group.^10^ These data represent an early version of VGER data and were imputed using MACH. This study included only individuals of European descent, as designated in the EHR^11,12^ and were genotyped on the Illumina Human660W-Quadv1_A platform.

*BLSA*

The Baltimore longitudinal study on Aging (BLSA) study^13^ is a population-based study aimed to evaluate contributors of healthy aging in the older population residing predominantly in the Baltimore-Washington DC area. Starting in 1958, participants are examined every one to four years depending on their age. Currently there are approximately 1100 active participants enrolled in the study. Blood samples were collected for DNA extraction, and genome-wide genotyping was completed for 1231 subjects using Illumina 550K. This study was restricted to subject of European descent with phenotype data.The BLSA has continuing approval from the Institutional Review Board (IRB) of Medstar Research Institute. 12 lead electrocardiograms were recorded with using standard electrode placements using MAC5000.

*BRIGHT*

The MRC BRIGHT study (<http://www.brightstudy.ac.uk/>) comprises 2000 severely hypertensive probands ascertained from families with multiplex affected sibships or as parent-offspring trios. Case ascertainment and phenotyping has been described previously. Briefly, cases have BP readings ≥150/100 mmHg based on one reading or ≥145/95 mmHg based on the mean of three readings. Twelve-lead ECG recordings (Siemens-Sicard 440; <http://www.brightstudy.ac.uk/info/sop04.html>), which produces an automated measurement of the P, QRS,T voltages as well as PR, QRS and QT intervals, were available for all subjects. All data were transferred from each recruitment centre by electronic modem to electrophysiologists from the West of Scotland Coronary Prevention Study (Professor Peter Macfarlane) for central reporting. All individuals included in the analysis were of white British ancestry (up to level of grandparents).

*CARL*

INGI CARL includes samples coming from isolated populations and belong to the ITALIAN NETWORK OF GENETIC ISOLATES (INGI). INGI CARL examined about 1200 subjects between 1998 and 2005 coming from a small village of the South of Italy situated in the extreme northern part of Puglia Region. Digital caliper measurements were made on scanned paper ECGs recorded at 25 mm/sec. Mortara instrument ELI 250 was used to obtain ECG measurements.

*CHS*

The Cardiovascular Health Study ([www.chs-nhlbi.org](http://www.chs-nhlbi.org)) is a prospective, longitudinal cohort study of risk factors for cardiovascular disease in the elderly, was begun in 1989 and included 4,925 self-described White participants. People 65 years of age or older were recruited from four field centers in the United States. The CHS study sample used in this analysis includes participants without clinically-recognized cardiovascular disease at baseline who described their race as White, consented to genetic testing, and had DNA available for genotyping. Study electrocardiograms were recorded using MAC PC ECG machines (Marquette Electronics, Milwaukee, Wisconsin) in all clinical centers. ECGs were initially processed in a central laboratory at the EPICORE Center (University of Alberta, Edmonton, Alberta, Canada) and during later phases of the study, at the EPICARE Center (Wake Forest University, Winston-Salem, North Carolina). All ECGs were visually inspected for technical errors and inadequate quality. PR interval was measured using the baseline ECG for eligible subjects. Initial ECG processing was done by the Dalhousie ECG program, and processing was later repeated with the 2001 version of the GE Marquette 12-SL program (GE Marquette, Milwaukee, Wisconsin).

*CROATIA-Korcula*

The CROATIA-Korcula study^14^ sampled Croatians from the Adriatic island of Korcula, between the ages of 18 and 88. The fieldwork was performed in 2007 in the eastern part of the island, targeting healthy volunteers from the town of Korčula and the villages of Lumbarda, Žrnovo and Račišće. ECG recording: Mortara ELI 350

*CROATIA-Split*

CROATIA-Split study^15^ sampled Croatians from the city of Split, between the ages 18 and 85. The samples analysed here were collected in 2008. ECG recording: Mortara ELI 350

*Decode*

The Icelandic study was approved by the Data Protection Commission of Iceland and the National Bioethics Committee of Iceland (VSN-15-058). Written informed consent was obtained from subjects with genotypes. Personal identifiers associated with medical information and blood samples were encrypted with a third-party encryption system as provided by the Data Protection Commission of Iceland. This analysis included all ECGs obtained and digitally stored between 2004 and 2008 at the Landspitali, National University Hospital, Reykjavik, the largest medical center and only tertiary referral center in Iceland, from individuals with genotypes at deCODE.^16^ The ECGs were recorded digitally with the Philips PageWriter Trim III and PageWriter 200 cardiographs and stored in the Philips TraceMasterVue ECG Management System. These ECGs were obtained in all hospital departments, from both inpatients and outpatients. Digitally measured ECG waveforms and parameters were extracted from the database for analysis. The Philips PageWriter Trim III PR interval measurement algorithm has been described previously and shown to fulfill industrial ECG measurement accuracy standards.^17^

*ERF*

The Erasmus Rucphen Family study^18^ is comprised of a family-based cohort embedded in the Genetic Research in Isolated Populations (GRIP) program in the southwest of the Netherlands. The aim of this program is to identify genetic risk factors for the development of complex disorders. In ERF, twenty-two families that had a large number of children baptized in the community church between 1850 and 1900 were identified with the help of detailed genealogical records. All living descendants of these couples, and their spouses, were invited to take part in the study. Comprehensive interviews, questionnaires, and examinations were completed at a research center in the area; approximately 3,200 individuals participated. Examinations included 12 lead ECG measurements. Electrocardiograms were recorded on ACTA electrocardiographs (ESAOTE, Florence, Italy) and digital measurements of the PR interval were made using the Modular ECG Analysis System (MEANS). Data collection started in June 2002 and was completed in February 2005. In the current analyses, 1874 participants for whom complete phenotypic, genotypic and genealogical information was available were studied. Electrocardiograms were recorded on ACTA electrocardiographs (ESAOTE, Florence, Italy) and digital measurements of the PR interval were made using the Modular ECG Analysis System (MEANS).

*FHS*

The Framingham Heart Study^19^ is a prospective, community-based cohort study to examine the risk factors and outcomes associated with cardiovascular disease. The study was initiated in 1948 with enrollment of the Original Cohort (n=5,048). In 1971 the Framingham Heart Study enrolled the children of the Original Cohort and their spouses in what is described as the Offspring Cohort (n=5,124). The Generation 3 Cohort, comprised of the grandchildren of the Original Cohort, was subsequently enrolled in 2002 (n=4,095). Original Cohort participants have undergone examinations every 2 years since enrollment, and Offspring and Generation 3 Cohort participants every 4 to 8 years. Evaluations consist of a standardized history and physical examination; review of clinical events; routine and selected biomarkers; diverse imaging; and genomic studies. A full listing of available phenotypic evaluations may be found at www.framinghamheartstudy.org.” The current study included individuals from Generation 1 (11th examination), Generation 2 (1st examination) and Generation 3 (1st examination). Paper electrocardiograms recorded on Marquette machines were scanned and digital caliper measurements were made using proprietary software (eResearchTechnology, generations 1 and 2) or using Rigel 1.7.2. (AMPS, LLC, New York, NY, USA, generation 3). The PR duration was measured from the P-onset to Q-onset in two cardiac cycles from lead II and averaged.

*FVG*

INGI FVG includes samples coming from isolated populations and belong to the ITALIAN NETWORK OF GENETIC ISOLATES (INGI). INGI FVG involved about 2000 subjects between 2008 and 2011 coming from six different villages located in the North-East of Italy in Friuli Venezia Giulia region. Digital caliper measurements were made on scanned paper ECGs recorded at 25 mm/sec. Mortara instrument ELI 250 was used to obtain ECG measurements.

*GHS*

The Gutenberg Heart Study (GHS)^20^ was implemented in 2007 to generated a contemporary cardiovascular risk score and assess global health status. It is designed as a single-center, population-based, prospective cohort study that enrolled 15,010 participants, aged 35 to 74 years randomly invited from the City of Mainz and the District Mainz-Bingen between 2007 and 2012. The sample was drawn with equal strata for sex, decades of age and residence (urban vs. rural). A large variety of non-invasive cardiovascular phenotypes has been assessed in a highly standardized 6-hour examination including a 12-lead surface ECG (GE Cardiosoft®) during baseline examination. All data sets were recorded electronically. Automatically generated ECG values were used for this study.

*HealthABC*

The Health Aging and Body Composition (Health ABC) Study is a NIA-sponsored cohort study of the factors that contribute to incident disability and the decline in function of healthier older persons, with a particular emphasis on changes in body composition in old age. Between 4/15/97 and 6/5/98 the Health ABC study has recruited 3,075 70-79 year old community-dwelling adults (41% African-American), who were initially free of mobility and activities of daily living disability. The key components of Health ABC include a baseline exam, annual follow-up clinical exams, and phone contacts every 6 months to identify major health events and document functional status between clinic visits. Provision has been made for banking of blood specimens and extracted DNA (HealthABC repository). ECG recording information: Marquette MAC PC.

*InCHIANTI*

The InCHIANTI study^21^ is a population-based epidemiological study aimed at evaluating the factors that influence mobility in the older population living in the Chianti region in Tuscany, Italy. The details of the study have been previously reported. Briefly, 1616 residents were selected from the population registry of Greve in Chianti (a rural area: 11,709 residents with 19.3% of the population greater than 65 years of age), and Bagno a Ripoli (Antella village near Florence; 4,704 inhabitants, with 20.3% greater than 65 years of age). The participation rate was 90% (n=1453), and the subjects ranged between 21-102 years of age. Illumina Infinium HumanHap 550K SNP arrays were used for genotyping. The study protocol was approved by the Italian National Institute of Research and Care of Aging Institutional Review and Medstar Research Institute (Baltimore, MD). A standard resting 12-lead electrocardiogram was performed.

*KORA*

The KORA study^22,23^ is a series of independent population-based epidemiological surveys of participants living in the city of Augsburg, Southern Germany, or the two adjacent counties. All survey participants are residents of German nationality identified through the registration office and aged between 25 and 74 years at recruitment. The baseline survey KORA S3 was conducted in the years 1994/95 and KORA S4 in 1999-2001. 3,006 participants from KORA S3 were reexamined in a 10-year follow-up (KORA F3) in the years 2004/05. Genome-wide data for the analysis of the length of the QRS interval is available for random subsets of 1,644 persons from KORA F3 and 1,814 study participants from KORA S4. In both studies, 12-lead resting electrocardiograms were recorded with digital recording systems (F3: Mortara Portrait, Mortara Inc., Milwaukee, USA, S4: Hörmann Bioset 9000, Hörmann Medizinelektronik,Germany).

*LIFELINES*

LifeLines^24^ is a multi-disciplinary prospective population-based cohort study examining in a unique three-generation design the health and health-related behaviours of 165,000 persons living in the North East region of The Netherlands. It employs a broad range of investigative procedures in assessing the biomedical, socio-demographic, behavioural, physical and psychological factors which contribute to the health and disease of the general population, with a special focus on multimorbidity and complex genetics. Details of the protocol have been described elsewhere (https://www.lifelines.nl/lifelines-research/news). Standard 12-lead electrocardiograms were recorded with CardioPerfect equipment (Cardio Control; currently Welch Allyn, Delft, The Netherlands) and digital measurements of the PR intervals were extracted.

*ORCADES*

The Orkney Complex Disease Study (ORCADES)^25^ is a family-based, cross-sectional study in the isolated Scottish archipelago of Orkney. Genetic diversity in this population is decreased compared to Mainland Scotland, consistent with the high levels of endogamy historically. Data for participants aged 18-100 years, from a subgroup of ten islands, were used for this analysis. Fasting blood samples were collected and over 300 health-related phenotypes and environmental exposures were measured in each individual. All participants gave informed consent and the study was approved by Research Ethics Committees in Orkney and Aberdeen. ECG recording: 10 second digital ECG

*MESA*

The Multi-Ethnic Study of Atherosclerosis (MESA)^26,27^ is a study of the characteristics of subclinical cardiovascular disease (disease detected non-invasively before it has produced clinical signs and symptoms) and the risk factors that predict progression to clinically overt cardiovascular disease or progression of the subclinical disease. The cohort is a diverse, population-based sample of 6,814 asymptomatic men and women aged 45-84. Approximately 38 percent of the recruited participants are white, 28 percent African-American, 22 percent Hispanic, and 12 percent Asian (predominantly of Chinese descent). Participants were recruited during 2000-2002 from 6 field centers across the U.S. (at Wake Forest University; Columbia University; Johns Hopkins University; the University of Minnesota; Northwestern University; and the University of California – Los Angeles). All underwent anthropomorphic measurement and extensive evaluation by questionnaires at baseline, followed by 4 subsequent examinations at intervals of approximately 2-4 years. Age and sex were self-reported. ECGs were recorded in the supine position after a period of rest. MESA ECG data were collected using GE MAC 1200 electrocardiographs. Digitally collected ECGs were transferred via phone lines to the MESA ECG center (EPICARE). The ECGs were automatically processed by use of GE Marquette 12-SL software (2001 version), after visual inspection of the recordings for quality. Further information can be found at: [http://www.ncbi.nlm.nih.gov/projects/gap/cgi-bin/ study.cgi?study_id=phs000209.v13.p3](http://www.ncbi.nlm.nih.gov/projects/gap/cgi-bin/%20study.cgi?study_id=phs000209.v13.p3)

*MICROS*

The MICROS study^28^ is part of the genomic health care program 'GenNova' and was carried out in three villages of the Val Venosta on the populations of Stelvio, Vallelunga and Martello. This study was an extensive survey carried out in South Tyrol (Italy) in the period 2001-2003. Study participants were volunteers from three isolated villages located in the Italian Alps, in a German-speaking region bordering with Austria and Switzerland. Due to geographical, historical and political reasons, the entire region experienced a prolonged period of isolation from surrounding populations. Genotyping was performed on just under 1,400 participants with 1,334 available for analysis after data cleaning. Information on participants health status was collected through a standardized questionnaire and clinical examinations, including digitized ECG measurements (Mortara Portrait, Mortara Inc., Milwaukee, USA). Individuals with identified U-waves were excluded from analysis. The PR interval is measured using the ECG Mortara Portrait (12 leads), Mortara Inc., Milwaukee, USA. Laboratory data were obtained from standard blood analyses.

*PREVEND*

The Prevention of REnal and Vascular ENd stage Disease (PREVEND) study^29^ is an ongoing prospective study investigating the natural course of increased levels of urinary albumin excretion and its relation to renal and cardiovascular disease. Inhabitants 28 to 75 years of age (n=85,421) in the city of Groningen, The Netherlands, were asked to complete a short questionnaire, 47% responded, and individuals were then selected with a urinary albumin concentration of at least 10 mg/L (n = 7,768) and a randomly selected control group with a urinary albumin concentration less than 10 mg/L (n = 3,395). Details of the protocol have been described elsewhere (www.prevend.org). Standard 12-lead electrocardiograms were recorded with CardioPerfect equipment (Cardio Control; currently Welch Allyn, Delft, The Netherlands) and digital measurements of the ST-T wave amplitudes were extracted.

*PROSPER*

PROSPER^30,31^ was a prospective multicenter randomized placebo-controlled trial to assess whether treatment with pravastatin diminishes the risk of major vascular events in elderly. Between December 1997 and May 1999, we screened and enrolled subjects in Scotland (Glasgow), Ireland (Cork), and the Netherlands (Leiden). Men and women aged 70-82 years were recruited if they had pre-existing vascular disease or increased risk of such disease because of smoking, hypertension, or diabetes. A total number of 5,804 subjects were randomly assigned to pravastatin or placebo. ECG recordings were made using a Burdick Eclipse 850i electrocardiograph and digital data were transmitted from the various recording sites to the ECG Core Lab in Glasgow Royal Infirmary for review and serial comparison. ECG measurements, including PR interval, were made by the University of Glasow ECG analysis program.

*RS*

The Rotterdam Study is a prospective population-based cohort study founded in 1990 in a suburb of Rotterdam, the Netherlands. The first cohort (RS I) consisted of 7,983 participants, aged 55 years and over. The second cohort (RS II) was recruited in 2000 with the same inclusion criteria and consisted of 3,011 participants. The third cohort (RS IIII) consisted of 3,932 participants, aged 45 years and over and was recruited in 2006. The rationale and design of the RS have been described in detail elsewhere.^32^ The Medical Ethics Committee of Erasmus Medical Center approved the study and written consent was obtained from all participants. Electrocardiograms were recorder on ACTA electrocardiographs (ESAOTE, Florence, Italy) and digital measurements of the QRS intervals were made using the Modular ECG Analysis System (MEANS).^33^

*SardiNIA*

The SardiNIA study has been previously described.^34-36^ Briefly, it is a large population-based study which consists of 6,921 individuals, males and females, ages 14-102 y, and representing >60% of the adult population of four villages in the Lanusei Valley of Sardinia. Samples have been characterized for several quantitative traits and medical conditions, including PR interval. The ECG was recorded on paper (ECG machine Cardiette 600) with the participant at rest.

*SHIP*

The Study of Health in Pomerania (SHIP) is a population-based project in West Pomerania, the north-east area of Germany.^37^ A sample from the population aged 20 to 79 years was drawn from population registries. First, the three cities of the region (with 17,076 to 65,977 inhabitants) and the 12 towns (with 1,516 to 3,044 inhabitants) were selected, and then 17 out of 97 smaller towns (with less than 1,500 inhabitants), were drawn at random. Second, from each of the selected communities, subjects were drawn at random, proportional to the population size of each community and stratified by age and gender. Only individuals with German citizenship and main residency in the study area were included. Finally, 7,008 subjects were sampled, with 292 persons of each gender in each of the twelve five-year age strata. In order to minimize drop-outs by migration or death, subjects were selected in two waves. The net sample (without migrated or deceased persons) comprised 6,267 eligible subjects. Selected persons received a maximum of three written invitations. In case of non-response, letters were followed by a phone call or by home visits if contact by phone was not possible. The SHIP-0 population finally comprised 4,308 participants (corresponding to a final response of 68.7%). Participants underwent a standardized, digital 12-lead ECG at rest in the supine position as a component of the cohort examination. Electrocardiograms were recorded using the Personal 120LD (Esaote, Genova, Italy).

*TwinsUK*

The TwinsUK cohort^38,39^ comprises unselected, mostly female volunteers ascertained from the general population through national media campaigns in the UK. Means and ranges of quantitative phenotypes in Twins UK were similar to an age-matched singleton sample from the general population. Zygosity was determined by standardized questionnaire and confirmed by DNA fingerprinting. Written informed consent was obtained from all participants before they entered the studies, which were approved by the local research ethics committee. ECG recordings: Cardiofax ECG-9020K (Nihon Kohden UK Ltd., Middlesex, UK; n=2726) or manually using a high-resolution digitizing board (GTCO CalComp Peripherals, USA; n=317). Phenotypic data of monozygotic twins (of which only one was genotyped) was averaged to optimize information.

*YFS*

The YFS is a population-based follow up-study started in 1980. The main aim of the YFS is to determine the contribution made by childhood lifestyle, biological and psychological measures to the risk of cardiovascular diseases in adulthood. In 1980, over 3,500 children and adolescents all around Finland participated in the baseline study. The follow-up studies have been conducted mainly with 3-year intervals. The latest 30-year follow-up study was conducted in 2010-11 (ages 33-49 years) with 2,063 participants. The study was approved by the local ethics committees (University Hospitals of Helsinki, Turku, Tampere, Kuopio and Oulu) and was conducted following the guidelines of the Declaration of Helsinki. All participants gave their written informed consent.

**African American ancestry**

Our study population of African ancestry contained 13,415 adults of African descent from ten cohorts: Health, Aging, and Body Composition Study (Health ABC n=1,054), Healthy Aging in Neighborhoods of Diversity across the Life Span Study (HANDLS, n=945), Women’s Health Initiative (WHI, n=4,149), ARIC study (n=2,391), CFS (n=267), JHS (n=1,962), MESA (n=1,627), Baltimore Longitudinal Study of Aging (BLSA, n=155), Bogalusa Heart Study (BHS, n=191), and the Cardiovascular Health Study (CHS, n=674). A GWAS of PR interval in this cohort was previously published, identifying six loci.^40^

**Supplementary Note 2: Acknowledgments and funding**

AGES-Reykjavik

This study has been funded by NIH contracts N01-AG-1-2100 and 271201200022C, the NIA Intramural Research Program, Hjartavernd (the Icelandic Heart Association), and the Althingi (the Icelandic Parliament). The study is approved by the Icelandic National Bioethics Committee, VSN: 00-063. The researchers are indebted to the participants for their willingness to participate in the study.

ARIC

The Atherosclerosis Risk in Communities Study is carried out as a collaborative study supported by National Heart, Lung, and Blood Institute contracts (HHSN268201100005C, HHSN268201100006C, HHSN268201100007C, HHSN268201100008C, HHSN268201100009C, HHSN268201100010C, HHSN268201100011C, and HHSN268201100012C), R01HL087641, R01HL59367 and R01HL086694; National Human Genome Research Institute contract U01HG004402; and National Institutes of Health contract HHSN268200625226C. The authors thank the staff and participants of the ARIC study for their important contributions. Infrastructure was partly supported by Grant Number UL1RR025005, a component of the National Institutes of Health and NIH Roadmap for Medical Research.

BioVU

U01 HG04603 (VGER: Vanderbilt Genome-Electronic Records Project), a node in the National Human Genome Research Institute–supported eMERGE-1 network. BioVU and the Synthetic Derivative were supported in part by Vanderbilt CTSA grant 1 UL1 RR024975

BLSA

The BLSA was supported by the Intramural Research Program of the NIH, National Institute on Aging.

BRIGHT

This work was supported by the Medical Research Council of Great Britain (grant number G9521010D); and the British Heart Foundation (grant number PG/02/128). A.F.D. was supportedby the British Heart Foundation (grant numbers RG/07/005/23633, SP/08/005/25115); and by the European Union Ingenious HyperCare Consortium: Integrated Genomics, Clinical Research, and Care in Hypertension (grant number LSHM-C7-2006-037093). The BRIGHT study is extremely grateful to all the patients who participated in the study and the BRIGHT nursing team. We would also like to thank the Barts Genome Centre staff for their assistance with this project. This work forms part of the research portfolio for the National Institute for Health Research Biomedical Research Centre at Barts and the NIHR Leicester Biomedical Research Centre, University of Leicester, UK.

CARL

The study was funded Regione FVG (L.26.2008). We thank Angela D’Eustacchio and Anna Morgan for technical support. We are very grateful to the municipal administrators for their collaboration on the projects and for logistic support. We would like to thank all participants to these studies.

CCAF

The Cleveland Clinic AF study is supported by the National Institutes of Health grants R01 HL 090620 and R01 HL 111314 to MKC, DVW, JB, and JDS, the NIH National Center for Research Resources for Case Western Reserve University and Cleveland Clinic Clinical and Translational Science Award UL1-RR024989, the Cleveland Clinic Department of Cardiovascular Medicine philanthropy research funds, and the Tomsich Atrial Fibrillation Research Fund.

CHS

This CHS research was supported by NHLBI contracts HHSN268201200036C, HHSN268200800007C, N01HC55222, N01HC85079, N01HC85080, N01HC85081, N01HC85082, N01HC85083, N01HC85086; and NHLBI grants U01HL080295, R01HL087652, R01HL105756, R01HL103612, R01HL120393, and R01HL130114 with additional contribution from the National Institute of Neurological Disorders and Stroke (NINDS). Additional support was provided through R01AG023629 from the National Institute on Aging (NIA). A full list of principal CHS investigators and institutions can be found at CHS-NHLBI.org. The provision of genotyping data was supported in part by the National Center for Advancing Translational Sciences, CTSI grant UL1TR000124, and the National Institute of Diabetes and Digestive and Kidney Disease Diabetes Research Center (DRC) grant DK063491 to the Southern California Diabetes Endocrinology Research Center. NS was supported by the Laughlin Family, and R01HL111089 and R01HL116747. The content is solely the responsibility of the authors and does not necessarily represent the official views of the National Institutes of Health.

CROATIA-Korcula

We would like to acknowledge the staff of several institutions in Croatia that supported the field work, including but not limited to The University of Split and Zagreb Medical Schools and the Croatian Institute for Public Health. We would like to acknowledge the invaluable contributions of the recruitment team in Korcula, the administrative teams in Croatia and Edinburgh and the participants. The SNP genotyping for the CROATIA-Korcula cohort was performed in Helmholtz Zentrum München, Neuherberg, Germany. CROATIA-Korcula was funded by the Medical Research Council UK, the Croatian Ministry of Science, Education and Sports (grant 216-1080315-0302), the European Union framework program 6 EUROSPAN project (contract no. LSHG-CT-2006-018947), the Croatian Science Foundation (grant 8875) and the Research Centre of Excellence in Personalized Medicine.

CROATIA-Split

We would like to acknowledge the staff of several institutions in Croatia that supported the field work, including but not limited to The University of Split Medical School and Croatian Institute for Public Health. The SNP genotyping was performed by AROS Applied Biotechnology, Aarhus, Denmark. CROATIA-Split was funded by the Medical Research Council UK, The Croatian Ministry of Science, Education and Sports (grant 216-1080315-0302), the European Union framework program 6 EUROSPAN project (contract no. LSHG-CT-2006-018947) and the Research Centre of Excellence in Personalized Medicine.

Decode

We gratefully thank all study participants, the research staff at deCODE and all our collaborators for an invaluable contribution to research.

ERF

The ERF study as a part of EUROSPAN (European Special Populations Research Network) was supported by European Commission FP6 STRP grant number 018947 (LSHG-CT-2006-01947) and also received funding from the European Community's Seventh Framework Programme (FP7/2007-2013)/grant agreement HEALTH-F4-2007-201413 by the European Commission under the programme “Quality of Life and Management of the Living Resources” of 5th Framework Programme (no. QLG2-CT-2002-01254). The ERF study was further supported by ENGAGE consortium and CMSB. High-throughput analysis of the ERF data was supported by joint grant from Netherlands Organisation for Scientific Research and the Russian Foundation for Basic Research (NWO-RFBR 047.017.043). We are grateful to all study participants and their relatives, general practitioners and neurologists for their contributions to the ERF study and to P Veraart for her help in genealogy, J Vergeer for the supervision of the laboratory work and P Snijders for his help in data collection.

FHS

Paper electrocardiograms recorded on Marquette machines were scanned and digital caliper measurements were made using proprietary software (eResearchTechnology, generations 1 and 2) or using Rigel 1.7.2. (AMPS, LLC, New York, NY, USA, generation 3). The PR duration was measured from the P-onset to Q-onset in two cardiac cycles from lead II and averaged.

FVG

The study was funded Regione FVG (L.26.2008). We thank Angela D’Eustacchio and Anna Morgan for technical support. We are very grateful to the municipal administrators for their collaboration on the projects and for logistic support. We would like to thank all participants to these studies.

GHS

This project has received funding from the European Research Council (ERC) under the European Union’s Horizon 2020 research and innovation programme (grant agreement No 648131), German Ministry of Research and Education (BMBF 01ZX1408A), and German Research Foundation Emmy Noether Programme (SCHN 1149/3-1). The Gutenberg Health Study is funded through the government of Rhineland-Palatinate („Stiftung Rheinland-Pfalz für Innovation“, contract AZ 961-386261/733), the research programs “Wissen schafft Zukunft” and “Center for Translational Vascular Biology (CTVB)” of the Johannes Gutenberg-University of Mainz, and its contract with Boehringer Ingelheim and PHILIPS Medical Systems, including an unrestricted grant for the Gutenberg Health Study. Philipp S. Wild is funded by the Federal Ministry of Education and Research (BMBF 01EO1503) and he is PI of the German Center for Cardiovascular Research (DZHK). We thank all study participants for their willingness to provide data for this research project and we are indebted to all coworkers for their enthusiastic commitment. Approval of the local ethics committee of Rhineland-Palatinate, Germany, reference no. 837.020.07 was obtained at all sites.

HAPI

We thank our Amish research volunteers for their long-standing partnership in research, and the research staff at the Amish Research Clinic for their hard work and dedication. We are supported by grants and contracts from the NIH including R01 AG18728 (Amish Longevity Study), R01 HL088119 (Amish Calcification Study), U01 GM074518-04 (PAPI Study), U01 HL072515-06 (HAPI Study), U01 HL084756 and NIH K12RR023250 (University of Maryland MCRDP), the University of Maryland General Clinical Research Center, grant M01 RR 16500, the Baltimore Veterans Administration Medical Center Geriatrics Research and Education Clinical Center and the Paul Beeson Physician Faculty Scholars in Aging Program.

HealthABC

This research was supported by NIA contracts N01AG62101, N01AG62103, and N01AG62106. The genome-wide association study was funded by NIA grant 1R01AG032098-01A1 to Wake Forest University Health Sciences and genotyping services were provided by the Center for Inherited Disease Research (CIDR). CIDR is fully funded through a federal contract from the National Institutes of Health to The Johns Hopkins University, contract number HHSN268200782096C. This research was supported in part by the Intramural Research Program of the NIH, National Institute on Aging.

InCHIANTI

The InCHIANTI study baseline (1998-2000) was supported as a "targeted project" (ICS110.1/RF97.71) by the Italian Ministry of Health and in part by the U.S. National Institute on Aging (Contracts: 263 MD 9164 and 263 MD 821336).

KORA

The KORA study was initiated and financed by the Helmholtz Zentrum München – German Research Center for Environmental Health, which is funded by the German Federal Ministry of Education and Research (BMBF) and by the State of Bavaria. Furthermore, KORA research was supported within the Munich Center of Health Sciences (MC-Health), Ludwig-Maximilians-Universität, as part of LMUinnovativ.

LIFELINES

The LifeLines Cohort Study, and generation and management of GWAS genotype data for the LifeLines Cohort Study is supported by the Netherlands Organization of Scientific Research NWO (grant 175.010.2007.006), the Economic Structure Enhancing Fund (FES) of the Dutch government, the Ministry of Economic Affairs, the Ministry of Education, Culture and Science, the Ministry for Health, Welfare and Sports, the Northern Netherlands Collaboration of Provinces (SNN), the Province of Groningen, University Medical Center Groningen, the University of Groningen, Dutch Kidney Foundation and Dutch Diabetes Research Foundation. Niek Verweij is supported by ICIN-NHI and Marie Sklodowska-Curie GF (call: H2020-MSCA-IF-2014, Project ID: 661395) and a NWO VENI grant (016.186.125). We thank Behrooz Alizadeh, Annemieke Boesjes, Marcel Bruinenberg, Noortje Festen, Ilja Nolte, Lude Franke, Mitra Valimohammadi for their help in creating the GWAS database, and Rob Bieringa, Joost Keers, René Oostergo, Rosalie Visser, Judith Vonk for their work related to data-collection and validation. The authors are grateful to the study participants, the staff from the LifeLines Cohort Study and Medical Biobank Northern Netherlands, and the participating general practitioners and pharmacists. LifeLines Scientific Protocol Preparation: Rudolf de Boer, Hans Hillege, Melanie van der Klauw, Gerjan Navis, Hans Ormel, Dirkje Postma, Judith Rosmalen, Joris Slaets, Ronald Stolk, Bruce Wolffenbuttel; LifeLines GWAS Working Group: Behrooz Alizadeh, Marike Boezen, Marcel Bruinenberg, Noortje Festen, Lude Franke, Pim van der Harst, Gerjan Navis, Dirkje Postma, Harold Snieder, Cisca Wijmenga, Bruce Wolffenbuttel. The authors wish to acknowledge the services of the LifeLines Cohort Study, the contributing research centres delivering data to LifeLines, and all the study participants.

ORCADES

ORCADES was supported by the Chief Scientist Office of the Scottish Government (CZB/4/276, CZB/4/710), the Royal Society, the MRC Human Genetics Unit, Arthritis Research UK and the European Union framework program 6 EUROSPAN project (contract no. LSHG-CT-2006-018947). DNA extractions were performed at the Wellcome Trust Clinical Research Facility in Edinburgh. We would like to acknowledge the invaluable contributions of Lorraine Anderson and the research nurses in Orkney, the administrative team in Edinburgh and the people of Orkney.

MESA

This research was supported by the Multi-Ethnic Study of Atherosclerosis (MESA) contracts HHSN2682015000031, N01-HC-95159, N01-HC-95160, N01-HC-95161, N01-HC-95162, N01-HC-95163, N01-HC-95164, N01-HC-95165, N01-HC-95166, N01-HC-95167, N01-HC-95168, N01-HC-95169 and by grants UL1-TR-000040, UL1-TR-001079, and UL1-RR-025005 from NCRR. Funding for MESA Family was provided by grants R01-HL-071205, R01-HL-071051, R01-HL-071250, R01-HL-071251, R01-HL-071252, R01-HL-071258, and R01-HL-071259, and by UL1-RR-025005 and UL1RR033176 from NCRR. Funding for MESA SHARe genotyping was provided by NHLBI Contract N02-HL-6-4278. The provision of genotyping data was supported in part by the National Center for Advancing Translational Sciences, CTSI grant UL1TR000124, and the National Institute of Diabetes and Digestive and Kidney Disease Diabetes Research Center (DRC) grant DK063491 to the Southern California Diabetes Endocrinology Research Center.

MICROS

This study was supported by the Ministry of Health of the Autonomous Province of Bolzano and the South Tyrolean Sparkasse Foundation.

PREVEND

PREVEND genetics is supported by the Dutch Kidney Foundation (Grant E033), the EU project grant GENECURE (FP-6 LSHM CT 2006 037697), the National Institutes of Health (grant 2R01LM010098), The Netherlands organization for health research and development (NWO-Groot grant 175.010.2007.006, NWO VENI grant 916.761.70, ZonMw grant 90.700.441), and the Dutch Inter University Cardiology Institute Netherlands (ICIN). N.Verweij is supported by Netherlands Heart Institute and Marie Sklodowska-Curie Global Fellowship (grant 661395).

PROSPER

The PROSPER study was supported by an investigator initiated grant obtained from Bristol-Myers Squibb. Prof. Dr. J. W. Jukema is an Established Clinical Investigator of the Netherlands Heart Foundation (grant 2001 D 032). Support for genotyping was provided by the seventh framework program of the European commission (grant 223004) and by the Netherlands Genomics Initiative (Netherlands Consortium for Healthy Aging grant 050-060-810).

RS

The Rotterdam Study (RS) is supported by the Erasmus Medical Center and Erasmus University Rotterdam; The Netherlands Organization for Scientific Research; The Netherlands Organization for Health Research and Development (ZonMw); the Research Institute for Diseases in the Elderly; The Netherlands Heart Foundation; the Ministry of Education, Culture and Science; the Ministry of Health Welfare and Sports; the European Commission; and the Municipality of Rotterdam. Support for genotyping was provided by The Netherlands Organization for Scientific Research (NWO) (175.010.2005.011, 911.03.012) and Research Institute for Diseases in the Elderly (RIDE). This study was supported by The Netherlands Genomics Initiative (NGI)/Netherlands Organization for Scientific Research (NWO) project nr. 050-060-810.

SardiNIA

We thank the many volnuteers who generously participated in this study, the Mayors and citizens of the Sardinian towns involved, the head of the Public Health Unit ASL4, and the province of Ogliastra for their volunteerism and cooperation. In addition, we are grateful to the Mayor and the administration in Lanusei for providing and furnishing the clinic site. We are grateful to the physicians Angelo Scuteri, Marco Orrù, Maria Grazia Pilia, Liana Ferreli, Francesco Loi, nurses Paola Loi, Monica Lai and Anna Cau who carried out participant physical exams; the recruitment personnel Susanna Murino; and Mariano Dei, Sandra Lai, Andrea Maschio, Fabio Busonero for genotyping. This research was supported by the Intramural Research Program of the NIH, National Institute on Aging with contracts N01-AG-1-2109 and HHSN271201100005C, and by grant FaReBio2011 “Farmaci e Reti Biotecnologiche di Qualità”.

SHIP

SHIP is part of the Community Medicine Research net of the University of Greifswald, Germany, which is funded by the Federal Ministry of Education and Research (grants no. 01ZZ9603, 01ZZ0103, and 01ZZ0403), the Ministry of Cultural Affairs as well as the Social Ministry of the Federal State of Mecklenburg-West Pomerania, and the network ‘Greifswald Approach to Individualized Medicine (GANI_MED)’ funded by the Federal Ministry of Education and Research (grant 03IS2061A). Genome-wide data have been supported by the Federal Ministry of Education and Research (grant no. 03ZIK012) and a joint grant from Siemens Healthcare, Erlangen, Germany and the Federal State of Mecklenburg- West Pomerania. The University of Greifswald is a member of the Caché Campus program of the InterSystems GmbH.

TwinsUK

The study was funded by the Wellcome Trust; European Community’s Seventh Framework Programme (FP7/2007-2013). The study also receives support from the National Institute for Health Research (NIHR) BioResource Clinical Research Facility and Biomedical Research Centre based at Guy's and St Thomas' NHS Foundation Trust and King's College London and the British Heart Foundation. Tim Spector is holder of an ERC Advanced Principal Investigator award. SNP Genotyping was performed by The Wellcome Trust Sanger Institute and National Eye Institute via NIH/CIDR. Statistical analyses were carried out on the Genetic Cluster Computer (http://www.geneticcluster.org) hosted by SURFsara and financially supported by the Netherlands Scientific Organization (NWO 480-05-003 PI: Posthuma) along with a supplement from the Dutch Brain Foundation and the VU University Amsterdam. Y.J. received funding for the project from the British Heart Foundation (PG/12/38/29615 and PG/06/094/21278).

YFS

The Young Finns Study has been financially supported by the Academy of Finland: grants 286284, 134309 (Eye), 126925, 121584, 124282, 129378 (Salve), 117787 (Gendi), and 41071 (Skidi); the Social Insurance Institution of Finland; Competitive State Research Financing of the Expert Responsibility area of Kuopio, Tampere and Turku University Hospitals (grant X51001); Juho Vainio Foundation; Paavo Nurmi Foundation; Finnish Foundation for Cardiovascular Research ; Finnish Cultural Foundation; Tampere Tuberculosis Foundation; Emil Aaltonen Foundation; Yrjö Jahnsson Foundation; Signe and Ane Gyllenberg Foundation; and Diabetes Research Foundation of Finnish Diabetes Association. The expert technical assistance in the statistical analyses by Irina Lisinen is gratefully acknowledged.

**Supplementary Note 3: Box**

**Cardiac Sodium Channel Genes**

*SCN5A* encodes Na_v_1.5, a subunit of a voltage-gated sodium channel highly expressed in the heart. *SCN5A* mutations impair channel function (e.g., alter inactivation and recovery rates),^41^ leading to clinical phenotypes, such as atrial fibrillation, long QT syndrome, and Brugada syndrome.^42,43^ Common SNPs have been associated with PR interval duration.^44^

*SCN10A* encodes the Na_v_1.8 sodium channel subunit, which was originally identified in dorsal root ganglion sensory neurons.^45^ *SCN10A* is also expressed in the heart and has been associated with PR interval.^16,44^ Specific blocking of transgenic human *SCN10A* in cultured cardiomyocytes (from mice whose endogenous gene was ablated) shortens action potentials, confirming involvement of *SCN10A* in heart rhythms.^46^

**Transcriptional regulator activity**

NKX2-5 is a homeodomain-containing transcription factor that is expressed early in the developing heart. NKX2-5, TBX5, and ID2 appear to be core transcription factors that together direct development of the cardiac conduction system.^28^ *NKX2-5* mutations can cause atrioventricular conduction block and heart malformations (e.g. atrial or ventricular septal defects, tetralogy of Fallot, hypoplastic left heart, and double outlet right ventricle).^47^ The gene has been associated with PR interval.^44^

*TBX5* and *TBX3* encode T-box-containing transcription factors involved in forming the cardiac conduction system and pacemaker, respectively. For example, mice with heterozygous deletions of *Tbx5* have prolonged PR and QRS durations.^48, 28^ On the other hand, overexpression of transgenic TBX3 in mice leads to ectopic atrial pacemakers.^49^ *TBX5* mutations cause Holt-Oram syndrome, a hereditary condition with upper limb malformations and congenital heart defects. Mutations in *TBX3* are found in ulnar-mammary syndrome, a disorder with abnormal development of forelimbs, apocrine glands, teeth, and (occasionally) the heart.^50,51^ Common variants in *TBX5*^16,44,52,53^ and *TBX3*^44^ have been associated with PR interval duration.

*TBX20* is essential for cardiac morphogenesis, maturation, and maintenance. TBX20 interacts directly with NKX2-5, TBX5, and other factors (e.g., GATA4 and GATA5), as shown in mice. Cardiac-specific knockdown of *Tbx20* in adult mice causes cardiomyopathy, heart failure, arrhythmias, and death (within 15 days), indicating that TBX20 is important for maintaining the heart.^54^ *TBX20* mutations are associated with atrial septal defects, tetralogy of Fallot, dilated cardiomyopathy, and other abnormalities.

MKLN1 is an intracellular protein involved in cytoskeleton dynamics and cell adhesion. Expressing tissues include the heart, skeletal muscles, brain, eyes, and kidneys. MKLN1 binds to proteins with diverse functions. ^55^ In developing hearts of mice, MKLN1 binds to TBX20b -- with staining in or around the interventricular septum and atrioventricular canal.^56^

*EOMES* is another member of the T-box family of transcription factors. It activates a network of regulators involved in mesodermal and endodermal differentiation in the early embryo.^57^ In one model, *TBX3* associates with a histone demethylase bound to an *Eomes* enhancer. EOMES binds to the *Mesp1* promoter to induce *Mesp1* expression, which in turn influences cardiovascular differentiation.^58^

*ID2* is in the ID family of transcriptional repressors, and promotes cell proliferation.^59^ Its promoter contains binding sites for TBX5 and NKX2-5, which synergistically activate *ID2*. As described above, NKX2-5, TBX5, and ID2 are key transcription factors for development of the cardiac conduction system.*^60,61^* Mice with *Id2* knockouts have widened QRS complexes.^60^

*MYOCD* encodes a nuclear protein expressed in the heart and aorta, as well as smooth muscle cells. It is a transcriptional co-activator of serum response factor (SRF) -- also a major transcription factor in heart development. With SRF, MYOCD activates BMP10 in a signaling pathway that stimulates maturation of the atria and ventricles. Therefore, *Myocd* null embryos have hypoplastic hearts (lethal).^62^

*SENP2* encodes a SUMO1-specific protease that deconjugates SUMOylated proteins. SUMOs (small ubiquitin-like modifiers) control various cell processes via post-translational modification of target proteins. Mouse pups with over-expressed, transgenic SENP2 often die in the first week of life (≥50%) -- with engorged atria and/or septal defects. Survivors have reduced heart and body weights, compared to wild-type.^63^ Several of the signaling molecules mentioned in this Box are regulated by SUMOylation (e.g., MYOCD, NKX2-5, ZFPM2).^64-66^

*ZFPM2* is a transcription co-factor (with GATA4, -5, and -6) that regulates formation of the heart and coronary vessels. When SUMOylated, ZFPM2 becomes a weaker transcriptional repressor.^67^ Mutations in *ZFPM2* are found in some individuals with congenital heart defects, such as tetralogy of Fallot and double outlet right ventricle.^68,69^

*MEIS1* encodes a homeobox transcription factor implicated in cardiac, hematopoietic, and neural development. Meis1-deficient mice have malformed cardiac outflow tracts with overriding aorta and ventricular septal defects.^70^ Common variation in *MEIS1* has been associated with PR interval duration.^44,52^

*MED13L* encodes a subunit of the Mediator complex, a transcriptional co-activator for most genes that are transcribed by RNA polymerase II. The Mediator complex forms a molecular bridge between transcription activators and RNA Pol II. *MED13L* is involved in early development of the heart and brain. Mutations in this gene have been shown to cause mental retardation with occasional accompanying cardiac defects (septal defects and/or transposition of the great arteries.^71^ One study found *MED13L* mutations in 3 out of 97 individuals with isolated dextro-looped transposition of the great arteries.^72^

*SOX5* encodes a member of the SRY-related HMG-box family of transcription factors involved in the regulation of embryonic development and determination of cell fate. Silencing of the *Drosophila* *Sox102F* gene (the ortholog of *SOX5*) results in severe cardiac dysfunction and structural defects, with disrupted WNT signal transduction.^73^ *Sox5* and *Sox6* double knockouts in mice lead to *in utero* heart failure and death (at around E16.5) -- perhaps related to severe skeletal abnormalities.^74^ *SOX5* has been associated with PR interval.^44^

*SKI* encodes the nuclear proto-oncogene protein homolog of avian sarcoma viral (v-ski) oncogene and is a repressor of TGF-β signaling. *SKI* appears to be anti-fibrotic in the heart, because overexpression in rat cardiac fibroblasts reduces collagen secretion and contractility.^75^ Morpholino knockdown of *skia* and *skib* in zebrafish results in incomplete looping of the heart and outflow tract malformations, as well as craniofacial defects.^76^ Mutations in *SKI* cause Shprintzen-Goldberg syndrome (with aortic root aneurysm).

*TLE3* is a transcriptional integrator of the PPARγ and Wnt pathways that has been shown to regulate adipogenesis.^77^ TLE3 has been shown to interact with TBX18, an important T-box gene in heart development.^78^ Variation in *TLE3* has been associated with rheumatioid arthritis.^79^

**Sarcomeric and extracellular matrix components**

*TTN* (Titin) is a sarcomeric protein that acts as a large molecular spring.^80^ *TTN* has 363 exons and gives rise to multiple isoforms with different spring properties, through alternative splicing.^81^ *TTN* mutations can cause cardiomyopathy^82^ and arrhythmogenic right ventricular dysplasia.^83^ A number of the other novel loci identified interact with *TTN* (such as *OBSCN* and CaMKii).

Ca^2+^/calmodulin-dependent protein kinase-II (CaMKii, a multi-subunit enzyme) has been shown to regulate diastolic stress in normal and failing hearts, via *TTN* phosphorylation.^84^ Active CaMKII enzymes are usually 12-mers. Subunits are encoded by the *CAMK2A*, *CAMK2B*, *CAMK2G*, and *CAMK2D* genes, which each give rise to different isoforms through alternative splicing (~40 total). *CAMK2D* is the most important CaMKII subunit in the heart.^85^ Expression of *CAMK2D* in left ventricular tissue was found to be increased in patients with dilated cardiomyopathy.^86^

*MYBPH* encodes the myosin binding protein H-like protein. *MYBPH*, like *TTN*, is associated with the thick filament in muscle and is expressed in ventricular Purkinje cells.^87^ It has been showed to play a role with cardiac contraction, and interacts with myosin, actin, and UBC9.^88^

OBSCN is a sarcomere protein with adhesion and signaling motifs, and it interacts with *TTN*.^89^ It appears to play a role in the assembly of myosin into sarcomeric A bands of striated muscle.

*MYH6* encodes the alpha myosin heavy chain, a cardiac myosin sarcomeric protein. MYH6 is a component of type II myosin. *MYH6* mutations are a cause of familial secundum-type atrial septal defects.^90^ A variant in *MYH6* has been associated with sick-sinus syndrome.^91^ *MYH6* variants have been associated with variation in resting heart rate.^16,92^ Heart rate *MYH6* variants have also been associated with and PR interval (*P* = 1.8 x 10^-5^).^16^

*FERMT2* (also known as kindlin-2) is a member of the family of genes that contain a FERM domain (four point one protein, ezrin, radixin, moesin).^93^ These proteins participate in connections between the extracellular matrix and the actin cytoskeleton. *FERMT2* is highly expressed in the mouse heart and is enriched at intercalated discs and in costameres of striated muscle. Homozygous disruption in mice causes embryonic death before formation of the heart. Morpholino knockdown in zebrafish leads to abnormal cardiac development and contractility.^94^ Silencing of the analogous gene in drosophila leads to disruped syncytium development and severe cardiomyopathy.^95^

*PDZRN3* contains a RING-finger motif in its N-terminal region, two PDZ domains in its central region, and a consensus-binding motif for PDZ domains at its C-terminus. *PDZRN3* is expressed in human heart and skeletal tissue and is required for the differentiation of cultured mouse skeletal myoblasts to myotubes (C2C12 cells).^96^ Overexpression of transgenic *Pdzrn3* in mouse endothelial cells leads to weak endothelial cell junctions and bleeding into cranial ventricles.^97^

**Signaling molecules**

CAV1 (with CAV2 and CAV3) are the main proteins in caveolae, which are cup-shaped membrane raft regions involved in signal transduction (and other functions).^98^ *Cav1*^-/-^ mice have lower left ventricle conduction velocities and higher risks for ventricular tachycardia, compared to wild-type. A mechanism appears to be loss of inhibition of SRC by CAV1, followed by reduced connexin 43 and impaired gap junctions.^99^ Common *CAV1* variants have been associated with PR interval duration.^16,44^

*WNT11* is a member of the Wnt family of secreted signaling proteins and has an important role in cardiogenesis.^100^ WNT11 (with WNT5a) is essential for second heart field progenitor development.^101^ WNT11 has been shown to be essential for the elongation of myocites, and acts as a directional cue for myocyte orientation.^102^ *Wnt11* and *Wnt5a* homozygous double knockout embryos have a single heart chamber with a central outflow tract (non-viable). *Wnt11* null mice have a small right ventricle and outflow tract. A common variant in *WNT11* was associated with PR interval duration.^44^

*SORBS1* is involved in the regulation of insulin-stimulated signaling and the control of glucose uptake. It is involved in cell adhesion, growth factor signaling, and cytoskeleton formation. It is primarily expressed in the heart, skeletal muscle. SORBS1 was shown to be down regulated in end stage heart failure patients.^103^ *SORBS1* variants have been associated with obesity and type 2 diabetes.^104^

**MISC**

*ARHGAP24* encodes Rho GTPase–activating protein 24. Rho GTPases are known to have effects on actin remodeling, cell polarity, and cell migration. A rare variant in *ARHGAP24* was shown to be associated with familial mitral valve prolapse.^105^ Common *ARHGAP24* variants have been associated with PR interval.^16,52,53^

*FRMD4B* encodes FERM domain-containing protein 4B. It is expressed ubiquitously in human tissues. An intronic variant in *FRMD4B* has been associated with heart failure.^106,107^

*FIGN* is a member of the AAA family of enzymes (ATPases associated with diverse cellular activities).^108^ *FIGN* appears to be strongly expressed in embryonic and adult hearts of mice. *Fign* mutant mice have anomalies involving the semicircular canals, eyes, and skeleton. A common *FIGN* variant has been associated with blood pressure in Asians.^109,110^ A variant in *FIGN* has been shown to reduce the risk of congenital heart disease in Han Chinease populations. ^111^

*LRCH1* encodes for a protein with a leucine-rich repeat and a calponin homology domain. Variants may be associated with susceptibility to knee osteoarthritis.^112,113^ Variants near *LRCH1* have been shown to be associated with PR duration.^114^

*NAV2* is a member of the neuron navigator gene family. *NAV2* is primarily expressed in the brain, kidney, and liver, and fetal heart.^115^ Studies in rats have shown that *NAV2* expression appears to play a role in the development of the embryonic nervous system.^115^

*PHLDB2* encodes pleckstrin homology-like domain family B member 2 and is involved in cell migration, possibly through interactions with cytoskeletal actins and microtubules.^116^ It is mostly highly expressed in the heart, kidney, and placenta.^117^

*MICU2* is a subunit of the mitochondrial calcium uniporter (MCU) -- a calcium channel on the inner mitochondrial membrane. Silencing of *MICU1* and/or *MICU2* impairs mitochondrial calcium uptake.^118^ By this mechanism, control of calcium influx by MICU1, MICU2, and MCU may contribute to regulation of oxidative metabolism of the heart.^119^ *MICU1* has been associated with myopathy with extrapyramidal signs, an autosomal recessive disorder, and it is likely *MICU2* would function in a similar way.^120^

*BNIP1* encodes BCL2 interacting protein 1. It is a member of the BCL2/adenovirus E1B 19 kd-interacting protein (BNIP) family. *BNIP1* functions as a component of the syntaxin-18 SNARE complex and regulates retrograde transport from the Golgi to the endoplasmic reticulum.^121^ Adenovirus E1B 19-kD protein protects against cell death induced by viral infection and certain external stimuli.^122^

**Supplementary Tables**

**Supplementary Table 1: Novel loci identified by transethnic and pleiotropic meta-analyses.** We combined GWAS results of Europeans and African Americans and identified an additional five loci associated with PR interval. To identify loci associated with atrioventricular conduction, we combined data on PR interval with association results of QRS duration (one novel locus), of RR interval (no novel loci), and of atrial fibrillation (two novel loci). Furthermore, we tested SNPs in DHSs only, adjusting the significance threshold accordingly, and found another four SNPs significantly associated with PR interval. Because some of the loci overlapped, these analyses led to 6 novel loci in total.

|  |  |  | |  | |  | |  | | **P-value** | | | | |
| --- | --- | --- | --- | --- | --- | --- | --- | --- | --- | --- | --- | --- | --- | --- |
| **Locus** | **SNP** | | **Chr** | | **Position** | | **Closest Gene** | | **Supporting Analysis** | | **DHS** | **AA-EA Joint** | **PR QRS concordant** | **PR AF concordant** |
| 45 | rs2030569 | | 3 | | 66496608 | | *SLC25A26* | | AA-EAJoint | | - | 2.539E-08 | - | - |
| 46 | rs3732733 | | 3 | | 71286767 | | *FOXP1* | | DHS; AA-EAJoint | | 1.247E-07 | 4.837E-08 | - | - |
| 47 | rs2970852 | | 4 | | 23430621 | | *PPARGC1A* | | DHS; PR-QRSConcordant | | 5.600E-08 | - | 7.547E-09 | - |
| 48 | rs11970286 | | 6 | | 118787067 | | *SLC35F1* | | AA-EAJoint; PR-AFConcordant | | - | 4.166E-08 | - | 8.862E-10 |
| 49 | rs4871397 | | 8 | | 124635197 | | *KLHL38* | | DHS; AA-EAJoint; PR-AFConcordant | | 1.123E-07 | 3.165E-10 | - | 3.356E-11 |
| 50 | rs8046873 | | 16 | | 81309433 | | *CDH13* | | AA-EAJoint | | - | 3.369E-09 | - | - |

**Supplementary Table 2: Association results of DHS, meta-analyses of PR and QRS, RR, and AF, and meta-analysis of PR in Europeans and African Americans.** Only novel associations are reported. Associations were reported for the transethnic analysis if *P* < 5 x 10^-8^. Associations were reported for DHS analyses which contained only 122,278 SNPs if the Bonferroni-corrected P-value threshold exceeded *P* < 4 x 10^-7^. However, each of these DHS loci overlaps with findings that were significant in either transethnic or pleiotropic analyses. For the cross-trait analyses, the significance threshold was set at *P* < 8.3 x 10^-9^, to correct for 6 tests.

|  | **DHS identified novel PR loci** | | | | | | | | | | | |
| --- | --- | --- | --- | --- | --- | --- | --- | --- | --- | --- | --- | --- |
| **Locus** | **SNP** | | **Chr** | **Position (bp)** | | **Closest Gene** | **CA** | **NCA** | **CAF** | **Beta** | **SE** | **P-value** |
| 1 | rs3732733 | | 3 | 71286767 | | FOXP1 | A | G | 0.078 | 1.259 | 0.238 | 1.247E-07 |
| 2 | rs2932970 | | 4 | 23818820 | | PPARGC1A-intronic | A | G | 0.248 | 0.707 | 0.134 | 1.383E-07 |
| 4 | rs4871397 | | 8 | 124635197 | | KLHL38 | G | C | 0.070 | 1.458 | 0.275 | 1.123E-07 |
|  |  | |  |  | |  |  |  |  |  |  |  |
|  |  | |  |  | |  |  |  |  |  |  |  |  |  |  |  |  |  |  |
|  | **AA and EA joint meta-analysis novel PR loci** | | | | | | | | | | | |  |  |  |  |  |  |  |
| **Locus** | **SNP** | | **Chr** | **Position (bp)** | | **Closest Gene** | **CA** | **NCA** | **CAF EA** | **Beta EA** | **SE EA** | **P-value EA** | **CAF AA** | **Beta AA** | **SE AA** | **P-value AA** | **Beta META** | **SE META** | **P-value META** |
| 1 | rs2030569 | | 3 | 66496608 | | SLC25A26 | G | A | 0.294 | -0.682 | 0.129 | 1.379E-07 | 0.221 | -0.960 | 0.388 | 0.014 | -0.715 | 0.128 | 2.539E-08 |
| 2 | rs3732733 | | 3 | 71369457 | | FOXP1 | A | G | 0.078 | 1.259 | 0.238 | 1.247E-07 | 0.180 | 0.913 | 0.417 | 0.029 | 1.171 | 0.215 | 4.837E-08 |
| 3 | rs11970286 | | 6 | 118787067 | | SLC35F1 | T | C | 0.462 | -0.619 | 0.116 | 8.180E-08 | 0.248 | -0.815 | 0.394 | 0.039 | -0.636 | 0.116 | 4.166E-08 |
| 4 | rs4871397 | | 8 | 124704378 | | KLHL38 | G | C | 0.070 | 1.458 | 0.275 | 1.123E-07 | 0.068 | 2.843 | 0.675 | 2.501E-05 | 1.671 | 0.266 | 3.165E-10 |
| 5 | rs8046873 | | 16 | 81309433 | | CDH13 | T | C | 0.091 | -1.036 | 0.223 | 3.393E-06 | 0.154 | -1.839 | 0.490 | 1.750E-04 | -1.279 | 0.216 | 3.369E-09 |
|  |  | |  |  | |  |  |  |  |  |  |  |  |  |  |  |  |  |  |
|  |  | |  |  | |  |  |  |  |  |  |  |  |  |  |  |  |  |  |
|  | **PR and QRS concordant meta-analysis novel PR loci** | | | | | | | | | | | |  |  |  |  |  |
| **Locus** | **SNP** | | **Chr** | **Position (bp)** | | **Closest Gene** | **CA** | **NCA** | **CAF PR** | **Beta PR** | **SE PR** | **P-value PR** | **CAF QRS** | **Beta QRS** | **SE QRS** | **P-value QRS** | **P-value META** |
| 1 | rs2970852 | | 4 | 23430621 | | PPARGC1A | T | C | 0.250 | 0.718 | 0.132 | 5.600E-08 | 0.743 | 0.210 | 0.078 | 0.007 | 7.547E-09 |
|  |  | |  |  | |  |  |  |  |  |  |  |  |  |  |  |  |
|  | **PR and AF concordant meta-analysis novel PR loci** | | | | | | | | | | | |  |  |  |  |  |
| **Locus** | **SNP** | | **Chr** | **Position (bp)** | | **Closest Gene** | **CA** | **NCA** | **CAF PR** | **Beta PR** | **SE PR** | **P-value PR** | **CAF AF** | **Beta AF** | **SE AF** | **P-value AF** | **P-value META** |
| 1 | rs283077 | | 6 | 118,676,372 | | SLC35F1 | T | G | 0.498 | 0.569 | 0.115 | 7.291E-07 | 0.498 | 0.073 | 0.020 | 2.076E-04 | 8.862E-10 |
| 2 | rs4871397 | | 8 | 124,704,378 | | KLHL38 | C | G | 0.927 | -1.458 | 0.275 | 1.122E-07 | 0.932 | -0.179 | 0.044 | 4.794E-05 | 3.356E-11 |
|  |  |  | | |
|  |  |  | | |
|  |  |  | | |

**Supplementary Figures**

**
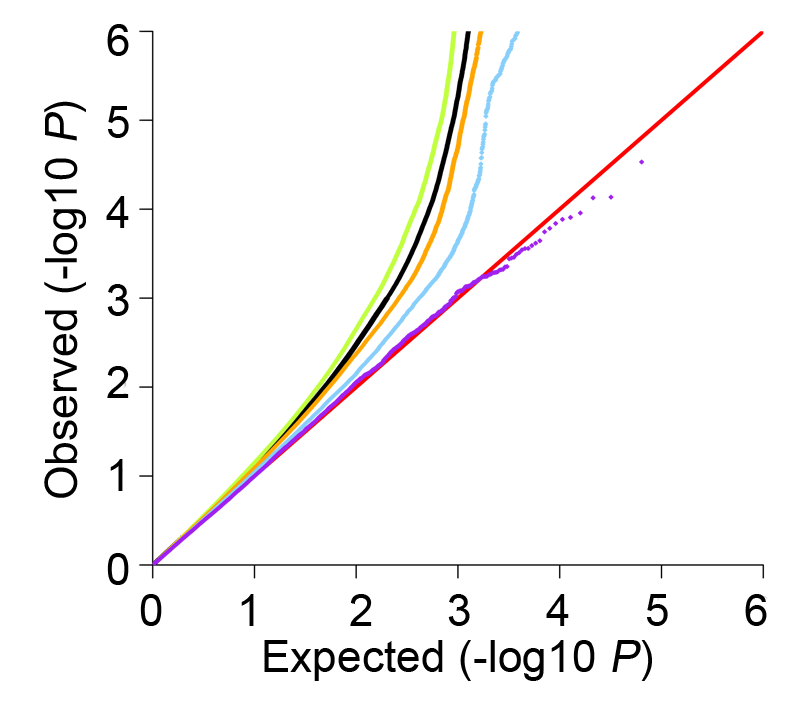
**

**Supplementary Figure 1: Quantile-quantile plot of PR-interval meta**

**analysis in 92,340 Europeans stratified by minor allele frequency.** The QQ-plot shows the observed versus expected distribution of P-values of meta-analysis results. The red line represents the null (expected) distribution, while the black dots show the observed distribution of all P-values. Green, orange, blue, and purple dots depict SNP with minor allele frequency above 20%, between 5% and 20%, between 1% and 5%, and below 1%, respectively. SNPs with allele frequencies above 1% show clear deviation from the null. Since PR interval is predicted to be highly polygenic, this deviation indicates many additional loci that may be involved in PR interval. The allele frequency bin below 1% has low power to detect associations and follows the null distribution. The overall genomic inflation factor lambda is 1.11. LD score regression shows that the majority of inflation is explained by the polygenicity of PR interval (see Supplementary Figure 3).

**Supplementary Figure 2: Regional association plots for all 44 loci associated with PR interval.** Each SNP is plotted with respect to its chromosomal location (x axis) and its P value (y axis on the left). The tall blue spikes indicate the recombination rate (y axis on the right) at that region of the chromosome**.** Red diamonds represents the index SNP (with the lowest P-value) for each locus. Smaller red-shaded diamonds correspond to SNPs in LD with the index SNP; color saturation indicates the degree of correlation with the index SNP. Independent SNPs in a locus are denoted by diamonds in gold, blue, purple, and green. Blue outlined squares mark non-synonymous SNPs. Green triangles depict association results of the African Americans meta-analysis,^53^ only SNPs with *P* < 0.1 are shown.

**
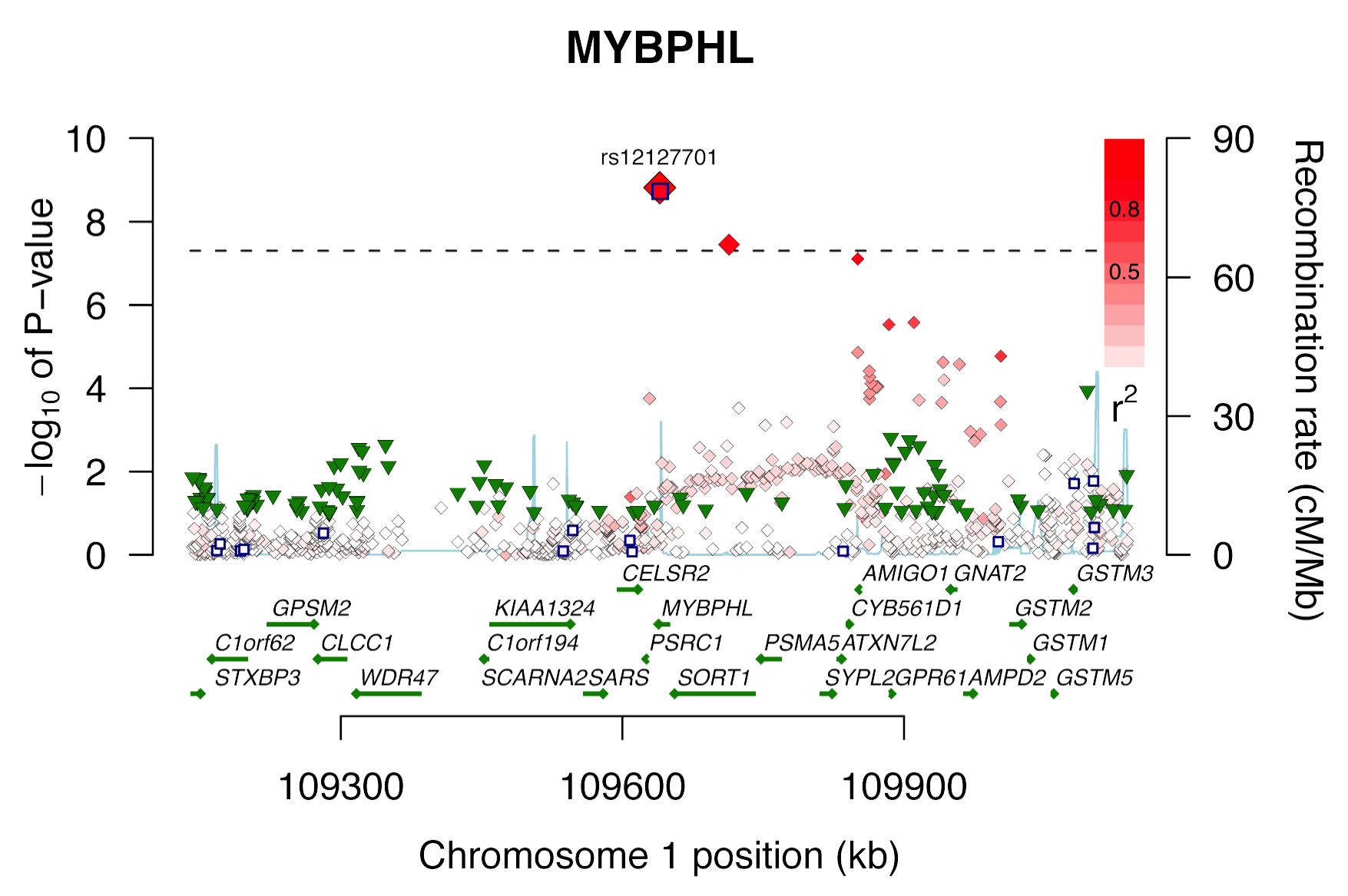
**

**
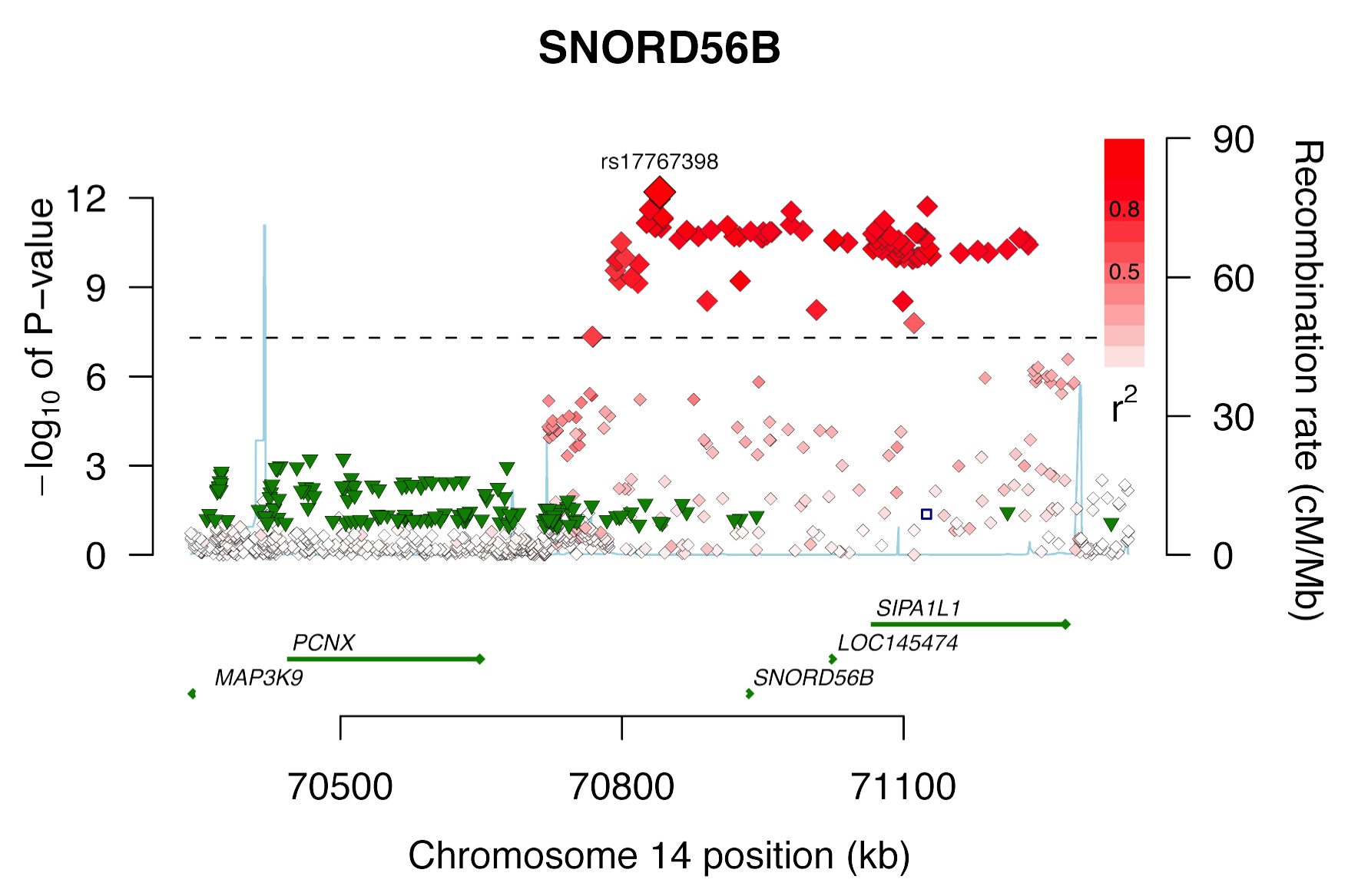
**

**
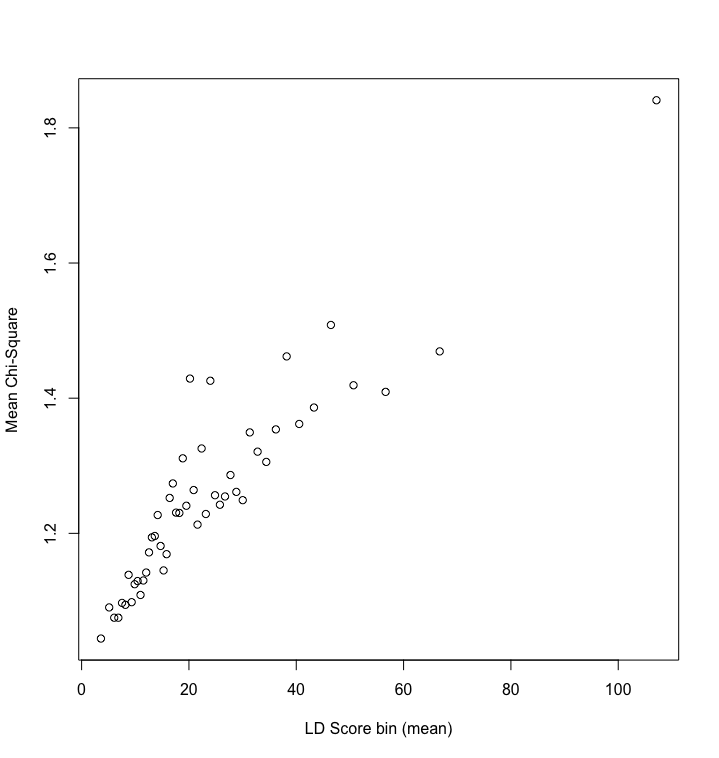
**

**Supplementary Figure 3: LD score plot of PR-interval association results in 92,340 Europeans**. LD scores were estimated using LDSC described by Bulik-Sullivan et al.139 and split into 50 bins. This plot shows the mean Chi-Square value for each bin. The LD score regression intercept and slope are 1.03 and 0.01, respectively. The h2 as calculated by LDSC is 0.15, and the inflation apart from true polygenicity is estimated at 0.12. Together, these results indicate that regions with more LD (i.e. stronger correlation between SNPs in the region) show an increased Chi-square statistic, and suggests that the increased genomic inflation factor lambda (1.11, see Supplementary Figure 1) is mostly caused by a true polygenic background of PR-interval.

**Supplementary Figure 4: Cardiac eQTL analysis.** Each SNP is plotted with respect to its chromosomal location (x axis) and its P value (y axis on the left) for association with transcript expression levels in cardiac left atrial tissue. The index SNP is bolded. The PR index SNP is listed but not bolded. Direction of effect is noted by direction of the triangle. Color denotes LD with index SNP, with red showing the highest LD. eQTL analysis of the MEIS1 and FAT1 locus in cardiac tissue show that the PR index SNP is in high LD with the top eQTL eSNP and likely represents the same signal.

**Supplementary Figure 5: Enrichment of signal in cardiac DHSes compared with DHSes from other tissue types.** Taking the full range of SNPs tested in our meta-analysis, we observe enrichment in DHSes of cardiovascular cell types for PR-associated SNPs above and below the threshold of genome-wide significance.

**Supplementary Figure 6: Mendelian Randomization analysis of PR SNPs and Pacemaker Implantation.** While prolongation of PR interval is causally related to pacemaker implantation, the estimate of the causal effect is small (estimate 0.0129, 95% CI 0.0048 – 0.0222), suggesting that acquired factors such as heart disease also play an important role.


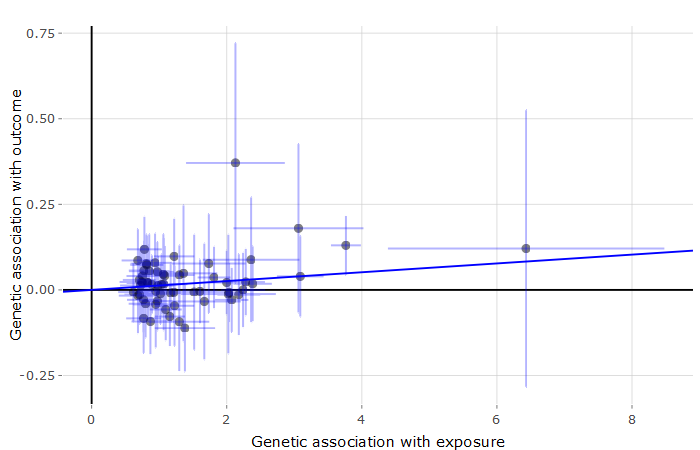


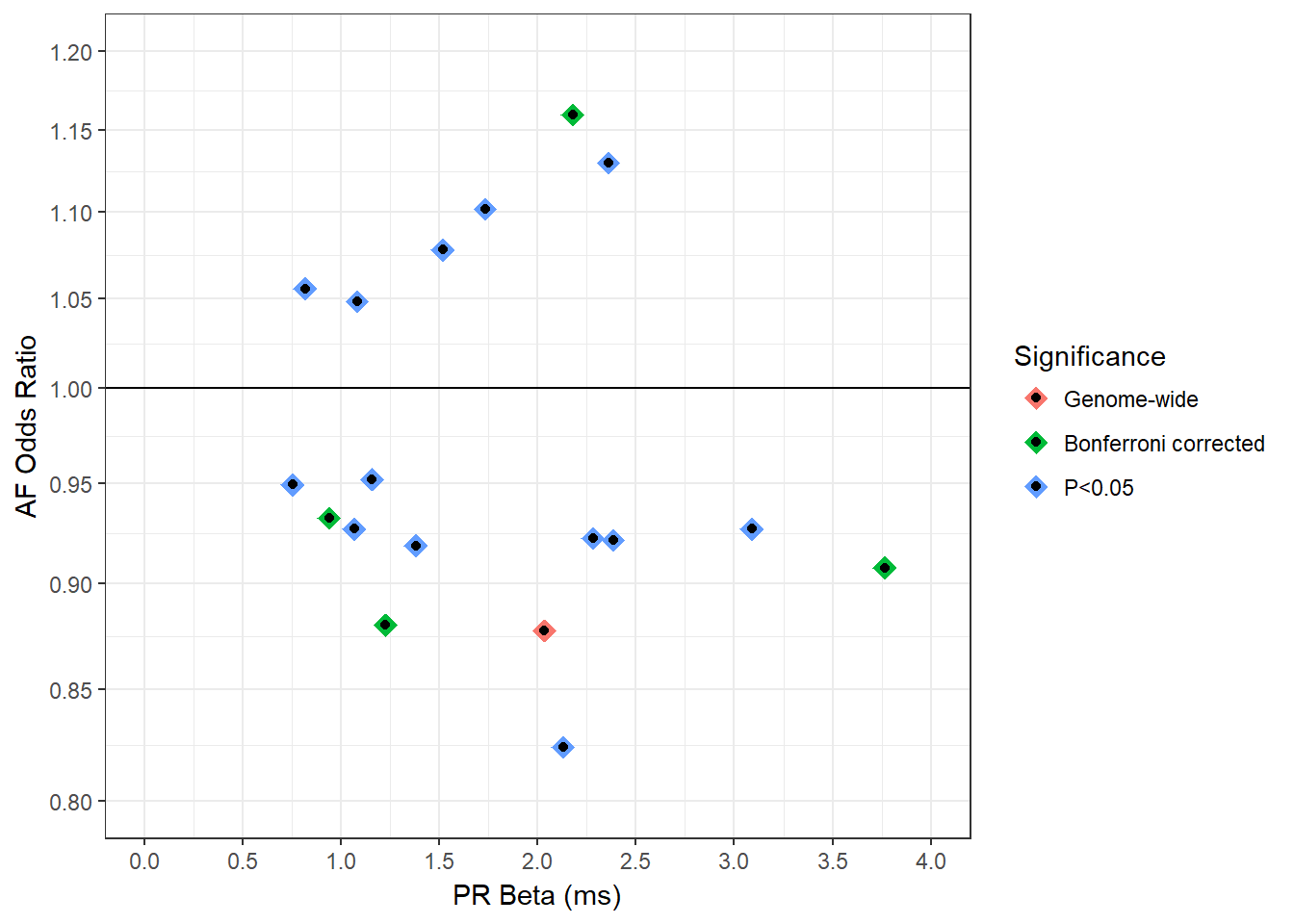
**Supplementary Figure 7: Overlapping genetic architecture between PR and QRS, PR and atrial fibrillation, PR and heart rate, and between PR interval in European and African Americans.** (a) The effect size of PR index SNPs on AF risk (y-axis) is plotted against PR interval duration (x-axis). Only PR SNPs with p<0.05 on AF risk are plotted. Intriguingly, SNPs with larger effects on PR interval also show greater effect on AF risk, but the effect can be either associated with higher or lower AF risk. (b) For all PR GWAS index SNPs in European samples, we plotted the effect sizes (betas) on PR interval (x-axis) and QRS duration (y-axis), where green squares and yellow squares represent PR index SNPs with P > 0.05 and P < 0.05 (but greater than 5 x 10^-8^) for QRS duration, respectively. We also plotted these effect sizes for QRS GWAS index SNPs found in a large GWAS of QRS duration, ^123^ where purple circles and grey circles depict QRS index SNPs with P > 0.05 and P < 0.05 (but greater than 5 x 10^-8^) for PR interval, respectively. Pink triangles denote SNPs that are significantly associated (*P* < 5 x 10^-8^) with both PR interval and QRS duration. Dark blue and light blue diamonds show SNPs that are not significant for either PR interval or QRS duration, but reach *P* < 5 x 10^-8^ in a joint meta-analysis of the two phenotypes, assuming concordant and discordant effects, respectively. This figure shows that while many of the PR-QRS SNP associations are concordant, there are a notable number of SNPs where the SNP effect on PR interval is discordant to its effects on QRS duration. (c) For all PR index SNPs in European samples, we plotted the effect sizes (betas) on PR interval (x-axis) and RR interval (y-axis). We also plotted these effect sizes for heart rate index SNPs found in a large GWAS of RR interval.^92^ where purple circles and grey circles depict RR index SNPs with P > 0.05 and P < 0.05 (but greater than 5 x 10^-8^) for PR interval, respectively. The colors and shapes on the graph represent p-values for PR and RR in the same manner as in (b) above. The vast majority of PR GWAS SNPs are not associated with RR, and vice versa. For the SNPs where there is at least nominal association with both phenotypes, the direction of effect can be concordant or discordant. Dark blue and light blue diamonds show SNPs that are not significant for either PR interval or RR interval, but reach genome-wide significance in a joint meta-analysis of the two phenotypes, assuming concordant and discordant effects, respectively.

a

b

a

c

d

(d) This plot shows the effect sizes (betas) of all genome-wide significant index SNPs observed in the European samples, as well as the four independent SNPs that were discovered in the combined meta-analysis comprising both European and African American samples (pink diamonds). Grey diamonds represent SNPs that show no association in the African American population (P > 0.05) and light blue diamonds represent SNPs that are nominally significant (P < 0.05) in African Americans. The effect sizes are highly correlated (Spearman’s correlation rho = 0.62, *P* = 6 x 10^-8^).

**Supplementary Figure 8: Influence of heart rate on genetic associations with PR interval.** (a) Graph of genetic effect size of the 61 non-redundant variants examined for association with PR interval among 8038 ARIC European descent participants after adjustment for age, gender, height, and body mass index, with (X-axis) and without (Y-axis) additional adjustment for RR interval on the surface electrocardiogram, a measure inversely proportional to heart rate. (b) P-value of the analyses of association of genetic variation with PR interval, with (X-axis) and without (Y-axis) adjustment for RR interval. (c) Beta, standard error, and P-value for the analyses in ARIC with and without adjustment for RR interval. Taken together, these data suggest that adjusting for heart rate does not importantly alter the associations identified for PR interval.

**a b**


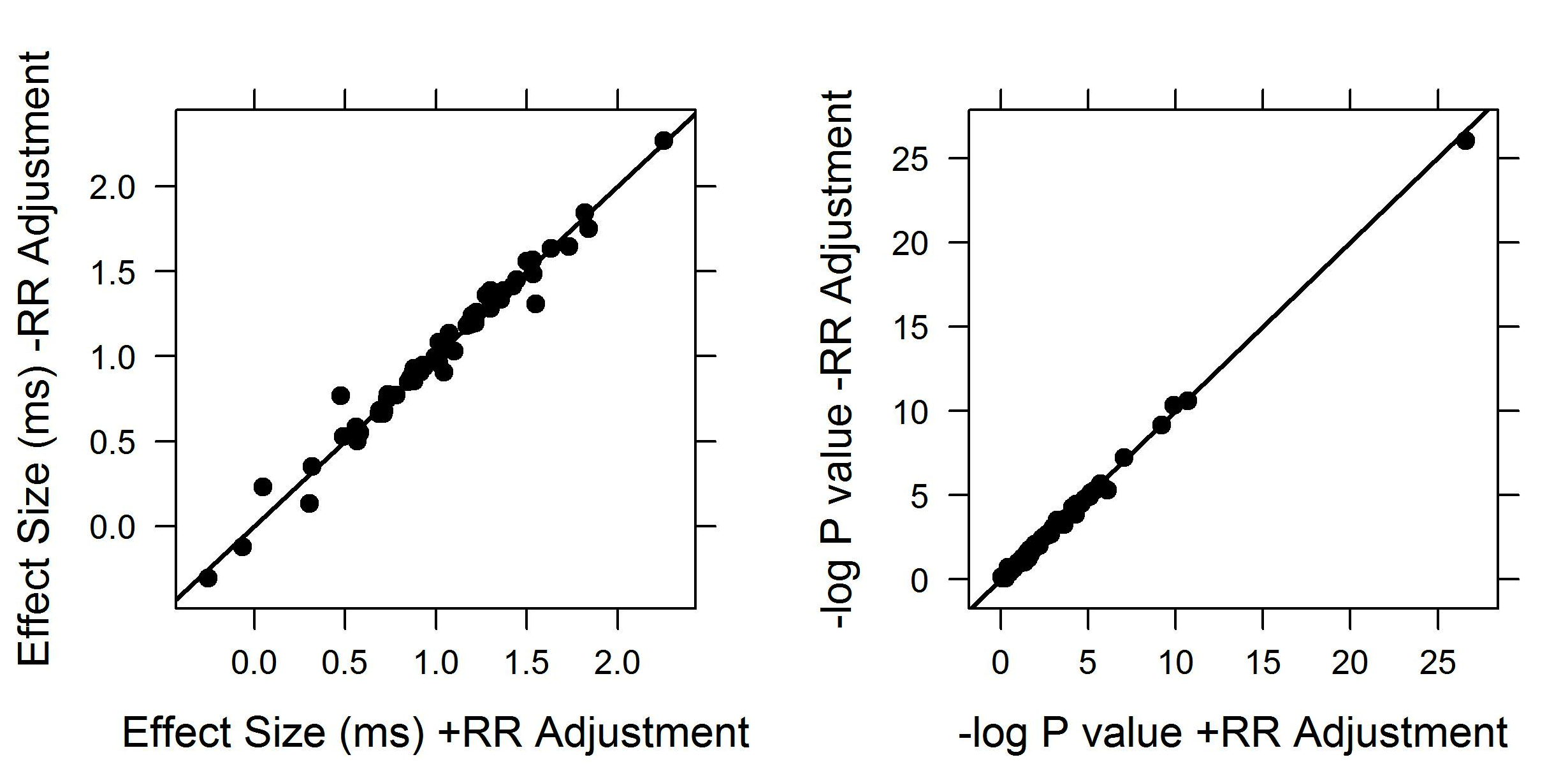


**c**

|  | **Without RR** | | | **With RR** | | |
| --- | --- | --- | --- | --- | --- | --- |
| **SNP** | **Beta** | **SE** | **P-value** | **Beta** | **SE** | **P-value** |
| rs10154914 | 0.850 | 0.187 | 5.440E-06 | 0.848 | 0.186 | 4.900E-06 |
| rs10748858 | 0.909 | 0.477 | 0.057 | 1.043 | 0.474 | 0.028 |
| rs10929536 | 1.350 | 0.313 | 1.690E-05 | 1.340 | 0.311 | 1.780E-05 |
| rs11067104 | 0.664 | 0.411 | 0.106 | 0.686 | 0.408 | 0.092 |
| rs11067228 | 1.560 | 0.385 | 5.310E-05 | 1.500 | 0.382 | 8.610E-05 |
| rs11067773 | 1.646 | 0.477 | 0.001 | 1.729 | 0.474 | 2.615E-04 |
| rs11264339 | 1.336 | 0.498 | 0.007 | 1.339 | 0.495 | 0.007 |
| rs11465506 | 1.311 | 0.778 | 0.092 | 1.547 | 0.772 | 0.045 |
| rs11708996 | 0.998 | 0.162 | 6.960E-10 | 0.993 | 0.160 | 6.450E-10 |
| rs11763856 | 0.528 | 0.389 | 0.174 | 0.489 | 0.386 | 0.205 |
| rs12127701 | 1.064 | 0.468 | 0.023 | 1.057 | 0.464 | 0.023 |
| rs12257568 | 0.550 | 0.349 | 0.115 | 0.580 | 0.346 | 0.094 |
| rs12359272 | 0.909 | 0.362 | 0.012 | 0.913 | 0.360 | 0.011 |
| rs12575413 | 0.503 | 0.591 | 0.395 | 0.567 | 0.587 | 0.334 |
| rs12673438 | 1.387 | 0.567 | 0.014 | 1.373 | 0.563 | 0.015 |
| rs12678719 | 2.270 | 0.506 | 7.250E-06 | 2.252 | 0.503 | 7.607E-06 |
| rs13018106 | 0.911 | 0.462 | 0.049 | 0.873 | 0.459 | 0.057 |
| rs13087058 | 1.086 | 0.373 | 0.004 | 1.049 | 0.370 | 0.005 |
| rs1372797 | -0.121 | 0.517 | 0.815 | -0.065 | 0.514 | 0.899 |
| rs1410059 | 1.198 | 0.576 | 0.038 | 1.215 | 0.572 | 0.034 |
| rs16858828 | 0.135 | 0.535 | 0.801 | 0.303 | 0.532 | 0.568 |
| rs17287293 | 1.030 | 0.224 | 4.730E-06 | 1.100 | 0.223 | 7.990E-07 |
| rs17446418 | 1.388 | 0.523 | 0.008 | 1.297 | 0.519 | 0.012 |
| rs17767398 | 1.335 | 0.430 | 0.002 | 1.356 | 0.427 | 0.002 |
| rs1805126 | 0.908 | 0.207 | 0.000 | 0.914 | 0.206 | 8.850E-06 |
| rs1873164 | 0.231 | 0.616 | 0.707 | 0.048 | 0.611 | 0.937 |
| rs1896312 | 1.180 | 0.248 | 2.050E-06 | 1.170 | 0.246 | 2.170E-06 |
| rs1984481 | 1.753 | 0.459 | 1.339E-04 | 1.840 | 0.456 | 5.440E-05 |
| rs2129561 | 1.244 | 0.368 | 0.001 | 1.197 | 0.366 | 0.001 |
| rs255292 | 1.282 | 0.332 | 1.119E-04 | 1.300 | 0.329 | 8.250E-05 |
| rs2585897 | 1.416 | 0.386 | 2.473E-04 | 1.423 | 0.383 | 2.075E-04 |
| rs2732860 | 0.354 | 0.396 | 0.372 | 0.316 | 0.393 | 0.422 |
| rs343849 | 0.773 | 0.186 | 0.000 | 0.778 | 0.184 | 2.500E-05 |
| rs35471 | 0.587 | 0.665 | 0.378 | 0.559 | 0.660 | 0.398 |
| rs365990 | 0.769 | 0.579 | 0.184 | 0.475 | 0.575 | 0.409 |
| rs3733409 | 0.680 | 0.550 | 0.216 | 0.712 | 0.546 | 0.192 |
| rs3807989 | 1.190 | 0.178 | 2.540E-11 | 1.190 | 0.177 | 2.060E-11 |
| rs3856447 | 0.876 | 0.292 | 0.003 | 0.860 | 0.290 | 0.003 |
| rs397637 | 1.139 | 0.507 | 0.025 | 1.070 | 0.503 | 0.034 |
| rs4430933 | 0.857 | 0.277 | 0.002 | 0.876 | 0.276 | 0.001 |
| rs4648819 | 1.255 | 0.556 | 0.024 | 1.226 | 0.552 | 0.026 |
| rs4901308 | 1.452 | 0.540 | 0.007 | 1.444 | 0.536 | 0.007 |
| rs6441111 | 0.937 | 0.444 | 0.035 | 0.935 | 0.441 | 0.034 |
| rs6489953 | 1.570 | 0.391 | 6.220E-05 | 1.530 | 0.389 | 8.560E-05 |
| rs6489974 | 1.361 | 0.375 | 2.838E-04 | 1.273 | 0.372 | 0.001 |
| rs652673 | 0.751 | 0.602 | 0.212 | 0.732 | 0.598 | 0.221 |
| rs6543191 | -0.304 | 0.458 | 0.506 | -0.256 | 0.454 | 0.573 |
| rs6599234 | 1.260 | 0.191 | 4.420E-11 | 1.220 | 0.190 | 1.280E-10 |
| rs6599250 | 1.030 | 0.096 | 9.070E-27 | 1.030 | 0.095 | 2.510E-27 |
| rs718426 | 0.664 | 0.312 | 0.034 | 0.709 | 0.310 | 0.022 |
| rs7372712 | 0.910 | 0.219 | 3.330E-05 | 0.882 | 0.218 | 5.050E-05 |
| rs7374138 | 0.950 | 0.175 | 5.680E-08 | 0.927 | 0.174 | 9.740E-08 |
| rs7538988 | 1.846 | 0.592 | 0.002 | 1.817 | 0.588 | 0.002 |
| rs7638853 | 0.929 | 0.545 | 0.088 | 0.877 | 0.541 | 0.105 |
| rs7729395 | 1.197 | 0.392 | 0.002 | 1.178 | 0.389 | 0.002 |
| rs881301 | 0.777 | 0.471 | 0.099 | 0.734 | 0.468 | 0.117 |
| rs900669 | 0.959 | 0.532 | 0.072 | 1.015 | 0.529 | 0.055 |
| rs904974 | 1.487 | 0.571 | 0.009 | 1.533 | 0.567 | 0.007 |
| rs922984 | 1.084 | 0.453 | 0.017 | 1.016 | 0.449 | 0.024 |
| rs9590974 | 0.683 | 0.349 | 0.050 | 0.688 | 0.346 | 0.047 |
| rs9826413 | 1.637 | 0.538 | 0.002 | 1.632 | 0.534 | 0.002 |

**Supplementary Figure 9: Manhattan plot of joint analyses including PR.** Overlay of PR results (in blue) and novel loci identified through joint analyses with African Americans (green), QRS duration (pink), and atrial fibrillation (yellow).

**
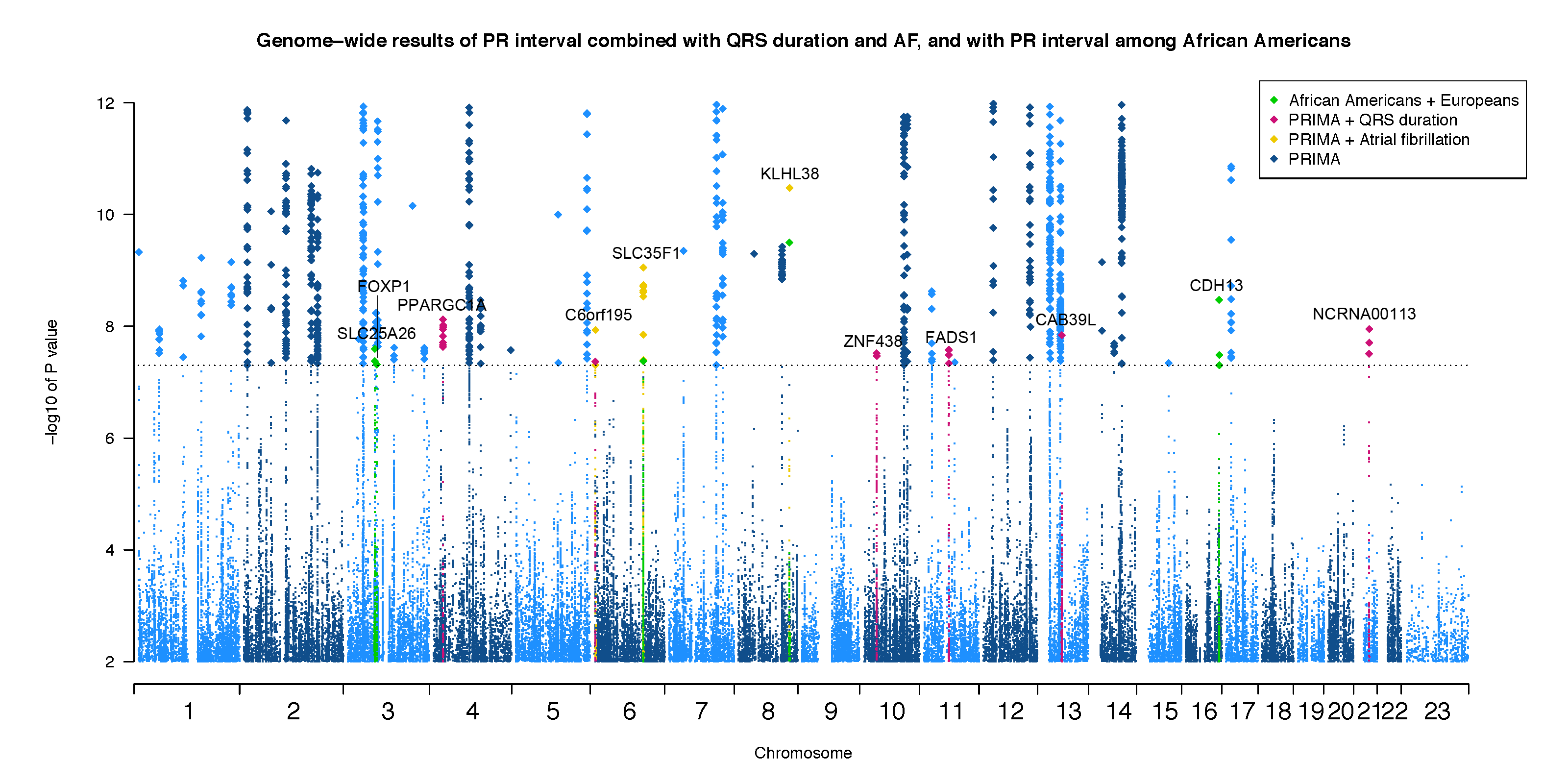
**

**References**

1. Harris, T.B. *et al.* Age, Gene/Environment Susceptibility-Reykjavik Study: multidisciplinary applied phenomics. *Am J Epidemiol* **165**, 1076-87 (2007).

2. Mitchell, B.D. *et al.* The genetic response to short-term interventions affecting cardiovascular function: rationale and design of the Heredity and Phenotype Intervention (HAPI) Heart Study. *Am Heart J* **155**, 823-8 (2008).

3. The Atherosclerosis Risk in Communities (ARIC) Study: design and objectives. The ARIC investigators. *Am J Epidemiol* **129**, 687-702 (1989).

4. Ritchie, M.D. *et al.* Robust replication of genotype-phenotype associations across multiple diseases in an electronic medical record. *Am J Hum Genet* **86**, 560-72 (2010).

5. Roden, D.M. *et al.* Development of a large-scale de-identified DNA biobank to enable personalized medicine. *Clin Pharmacol Ther* **84**, 362-9 (2008).

6. Pulley, J., Clayton, E., Bernard, G.R., Roden, D.M. & Masys, D.R. Principles of human subjects protections applied in an opt-out, de-identified biobank. *Clin Transl Sci* **3**, 42-8 (2010).

7. Ramirez, A.H. *et al.* Modulators of normal electrocardiographic intervals identified in a large electronic medical record. *Heart Rhythm* **8**, 271-7 (2011).

8. Ritchie, M.D. *et al.* Genome- and phenome-wide analyses of cardiac conduction identifies markers of arrhythmia risk. *Circulation* **127**, 1377-85 (2013).

9. McCarty, C.A. *et al.* The eMERGE Network: a consortium of biorepositories linked to electronic medical records data for conducting genomic studies. *BMC Med Genomics* **4**, 13 (2011).

10. Turner, S. *et al.* Quality control procedures for genome-wide association studies. *Curr Protoc Hum Genet* **Chapter 1**, Unit1 19 (2011).

11. Hall, J.B., Dumitrescu, L., Dilks, H.H., Crawford, D.C. & Bush, W.S. Accuracy of administratively-assigned ancestry for diverse populations in an electronic medical record-linked biobank. *PLoS One* **9**, e99161 (2014).

12. Dumitrescu, L. *et al.* Assessing the accuracy of observer-reported ancestry in a biorepository linked to electronic medical records. *Genet Med* **12**, 648-50 (2010).

13. Shock, N.W. *et al.* Normal Human Aging: The Baltimore Longitudinal Study on Aging. (NIH Publication, 1984).

14. Zemunik, T. *et al.* Genome-wide association study of biochemical traits in Korcula Island, Croatia. *Croat Med J* **50**, 23-33 (2009).

15. Rudan, I. *et al.* "10001 Dalmatians:" Croatia launches its national biobank. *Croat Med J* **50**, 4-6 (2009).

16. Holm, H. *et al.* Several common variants modulate heart rate, PR interval and QRS duration. *Nature genetics* **42**, 117-22 (2010).

17. Lindauer, J., Gregg, R., Helfenbein, E., Shao, M. & Zhou, S. Global QT measurements in the Philips 12-lead algorithm. *Journal of Electrocardiology* **38**, 90-90 (2005).

18. Pardo, L.M., MacKay, I., Oostra, B., van Duijn, C.M. & Aulchenko, Y.S. The effect of genetic drift in a young genetically isolated population. *Ann Hum Genet* **69**, 288-95 (2005).

19. Feinleib, M., Kannel, W.B., Garrison, R.J., McNamara, P.M. & Castelli, W.P. The Framingham Offspring Study. Design and preliminary data. *Prev Med* **4**, 518-25 (1975).

20. Schnabel, R.B., Johannsen, S.S., Wild, P.S. & Blankenberg, S. [Prevalence and risk factors of atrial fibrillation in Germany : data from the Gutenberg Health Study]. *Herz* **40**, 8-15 (2015).

21. Ferrucci, L. *et al.* Subsystems contributing to the decline in ability to walk: bridging the gap between epidemiology and geriatric practice in the InCHIANTI study. *J Am Geriatr Soc* **48**, 1618-25 (2000).

22. Holle, R., Happich, M., Lowel, H., Wichmann, H.E. & Group, M.K.S. KORA--a research platform for population based health research. *Gesundheitswesen* **67 Suppl 1**, S19-25 (2005).

23. Damin, D.C. *et al.* Atypical mycobacterial infection of the rectum. *Endoscopy* **37**, 788 (2005).

24. Scholtens, S. *et al.* Cohort Profile: LifeLines, a three-generation cohort study and biobank. *Int J Epidemiol* **44**, 1172-80 (2015).

25. McQuillan, R. *et al.* Runs of homozygosity in European populations. *Am J Hum Genet* **83**, 359-72 (2008).

26. Bild, D.E. *et al.* Multi-Ethnic Study of Atherosclerosis: objectives and design. *Am J Epidemiol* **156**, 871-81 (2002).

27. Rasmussen-Torvik, L.J. *et al.* Fasting glucose GWAS candidate region analysis across ethnic groups in the Multiethnic Study of Atherosclerosis (MESA). *Genet Epidemiol* **36**, 384-91 (2012).

28. Pattaro, C. *et al.* The genetic study of three population microisolates in South Tyrol (MICROS): study design and epidemiological perspectives. *BMC Med Genet* **8**, 29 (2007).

29. Hillege, H.L. *et al.* Urinary albumin excretion predicts cardiovascular and noncardiovascular mortality in general population. *Circulation* **106**, 1777-82 (2002).

30. Macfarlane, P.W. *et al.* The incidence and risk factors for new onset atrial fibrillation in the PROSPER study. *Europace* **13**, 634-9 (2011).

31. Shepherd, J. *et al.* Pravastatin in elderly individuals at risk of vascular disease (PROSPER): a randomised controlled trial. *Lancet* **360**, 1623-30 (2002).

32. Hofman, A. *et al.* The Rotterdam Study: 2012 objectives and design update. *Eur J Epidemiol* **26**, 657-86 (2011).

33. van Bemmel, J.H., Kors, J.A. & van Herpen, G. Methodology of the modular ECG analysis system MEANS. *Methods Inf Med* **29**, 346-53 (1990).

34. Pilia, G. *et al.* Heritability of cardiovascular and personality traits in 6,148 Sardinians. *PLoS Genet* **2**, e132 (2006).

35. Naitza, S. *et al.* A genome-wide association scan on the levels of markers of inflammation in Sardinians reveals associations that underpin its complex regulation. *PLoS Genet* **8**, e1002480 (2012).

36. Sidore, C. *et al.* Genome sequencing elucidates Sardinian genetic architecture and augments association analyses for lipid and blood inflammatory markers. *Nat Genet* **47**, 1272-81 (2015).

37. Volzke, H. *et al.* Cohort profile: the study of health in Pomerania. *Int J Epidemiol* **40**, 294-307 (2011).

38. Spector, T.D. & Williams, F.M. The UK Adult Twin Registry (TwinsUK). *Twin Res Hum Genet* **9**, 899-906 (2006).

39. Andrew, T. *et al.* Are twins and singletons comparable? A study of disease-related and lifestyle characteristics in adult women. *Twin Res* **4**, 464-77 (2001).

40. Butler, A.M. *et al.* Novel loci associated with PR interval in a genome-wide association study of 10 African American cohorts. *Circ Cardiovasc Genet* **5**, 639-46 (2012).

41. Wang, D.W., Yazawa, K., George, A.L., Jr. & Bennett, P.B. Characterization of human cardiac Na+ channel mutations in the congenital long QT syndrome. *Proceedings of the National Academy of Sciences of the United States of America* **93**, 13200-5 (1996).

42. Bezzina, C. *et al.* A single Na(+) channel mutation causing both long-QT and Brugada syndromes. *Circulation research* **85**, 1206-13 (1999).

43. Darbar, D. *et al.* Cardiac sodium channel (SCN5A) variants associated with atrial fibrillation. *Circulation* **117**, 1927-35 (2008).

44. Pfeufer, A. *et al.* Genome-wide association study of PR interval. *Nature genetics* **42**, 153-9 (2010).

45. Akopian, A.N., Sivilotti, L. & Wood, J.N. A tetrodotoxin-resistant voltage-gated sodium channel expressed by sensory neurons. *Nature* **379**, 257-62 (1996).

46. Yang, T. *et al.* Blocking Scn10a channels in heart reduces late sodium current and is antiarrhythmic. *Circulation research* **111**, 322-32 (2012).

47. Clark, K.L., Yutzey, K.E. & Benson, D.W. Transcription factors and congenital heart defects. *Annual review of physiology* **68**, 97-121 (2006).

48. Mori, A.D. *et al.* Tbx5-dependent rheostatic control of cardiac gene expression and morphogenesis. *Developmental biology* **297**, 566-86 (2006).

49. Hoogaars, W.M. *et al.* Tbx3 controls the sinoatrial node gene program and imposes pacemaker function on the atria. *Genes & development* **21**, 1098-112 (2007).

50. Bamshad, M. *et al.* Mutations in human TBX3 alter limb, apocrine and genital development in ulnar-mammary syndrome. *Nature genetics* **16**, 311-5 (1997).

51. Meneghini, V., Odent, S., Platonova, N., Egeo, A. & Merlo, G.R. Novel TBX3 mutation data in families with ulnar-mammary syndrome indicate a genotype-phenotype relationship: mutations that do not disrupt the T-domain are associated with less severe limb defects. *European journal of medical genetics* **49**, 151-8 (2006).

52. Smith, J.G. *et al.* Genome-wide association studies of the PR interval in African Americans. *PLoS genetics* **7**, e1001304 (2011).

53. Butler, A.M. *et al.* Novel loci associated with PR interval in a genome-wide association study of 10 African American cohorts. *Circulation. Cardiovascular genetics* **5**, 639-46 (2012).

54. Shen, T. *et al.* Tbx20 regulates a genetic program essential to adult mouse cardiomyocyte function. *The Journal of clinical investigation* **121**, 4640-54 (2011).

55. Kim, K.H., Hong, S.K., Hwang, K.Y. & Kim, E.E. Structure of mouse muskelin discoidin domain and biochemical characterization of its self-association. *Acta Crystallogr D Biol Crystallogr* **70**, 2863-74 (2014).

56. Debenedittis, P., Harmelink, C., Chen, Y., Wang, Q. & Jiao, K. Characterization of the novel interaction between muskelin and TBX20, a critical cardiogenic transcription factor. *Biochemical and biophysical research communications* **409**, 338-43 (2011).

57. Kartikasari, A.E. *et al.* The histone demethylase Jmjd3 sequentially associates with the transcription factors Tbx3 and Eomes to drive endoderm differentiation. *The EMBO journal* **32**, 1393-408 (2013).

58. van den Ameele, J. *et al.* Eomesodermin induces Mesp1 expression and cardiac differentiation from embryonic stem cells in the absence of Activin. *EMBO reports* **13**, 355-62 (2012).

59. Iavarone, A., Garg, P., Lasorella, A., Hsu, J. & Israel, M.A. The helix-loop-helix protein Id-2 enhances cell proliferation and binds to the retinoblastoma protein. *Genes Dev* **8**, 1270-84 (1994).

60. Moskowitz, I.P. *et al.* A molecular pathway including Id2, Tbx5, and Nkx2-5 required for cardiac conduction system development. *Cell* **129**, 1365-76 (2007).

61. Lim, J.Y., Kim, W.H., Kim, J. & Park, S.I. Induction of Id2 expression by cardiac transcription factors GATA4 and Nkx2.5. *Journal of cellular biochemistry* **103**, 182-94 (2008).

62. Huang, J. *et al.* Myocardin regulates BMP10 expression and is required for heart development. *J Clin Invest* **122**, 3678-91 (2012).

63. Kim, E.Y. *et al.* Enhanced desumoylation in murine hearts by overexpressed SENP2 leads to congenital heart defects and cardiac dysfunction. *Journal of molecular and cellular cardiology* **52**, 638-49 (2012).

64. Wang, J. *et al.* Myocardin sumoylation transactivates cardiogenic genes in pluripotent 10T1/2 fibroblasts. *Molecular and cellular biology* **27**, 622-32 (2007).

65. Wang, J., Zhang, H., Iyer, D., Feng, X.H. & Schwartz, R.J. Regulation of cardiac specific nkx2.5 gene activity by small ubiquitin-like modifier. *The Journal of biological chemistry* **283**, 23235-43 (2008).

66. Costa, M.W. *et al.* Complex SUMO-1 regulation of cardiac transcription factor Nkx2-5. *PloS one* **6**, e24812 (2011).

67. Perdomo, J., Jiang, X.M., Carter, D.R., Khachigian, L.M. & Chong, B.H. SUMOylation regulates the transcriptional repression activity of FOG-2 and its association with GATA-4. *PloS one* **7**, e50637 (2012).

68. De Luca, A. *et al.* New mutations in ZFPM2/FOG2 gene in tetralogy of Fallot and double outlet right ventricle. *Clinical genetics* **80**, 184-90 (2011).

69. Tan, Z.P., Huang, C., Xu, Z.B., Yang, J.F. & Yang, Y.F. Novel ZFPM2/FOG2 variants in patients with double outlet right ventricle. *Clinical genetics* **82**, 466-71 (2012).

70. Stankunas, K. *et al.* Pbx/Meis deficiencies demonstrate multigenetic origins of congenital heart disease. *Circulation research* **103**, 702-9 (2008).

71. Adegbola, A. *et al.* Redefining the MED13L syndrome. *Eur J Hum Genet* **23**, 1308-17 (2015).

72. Muncke, N. *et al.* Missense mutations and gene interruption in PROSIT240, a novel TRAP240-like gene, in patients with congenital heart defect (transposition of the great arteries). *Circulation* **108**, 2843-50 (2003).

73. Li, A. *et al.* Silencing of the Drosophila ortholog of SOX5 in heart leads to cardiac dysfunction as detected by optical coherence tomography. *Human molecular genetics* **22**, 3798-806 (2013).

74. Smits, P. *et al.* The transcription factors L-Sox5 and Sox6 are essential for cartilage formation. *Developmental cell* **1**, 277-90 (2001).

75. Cunnington, R.H. *et al.* Antifibrotic properties of c-Ski and its regulation of cardiac myofibroblast phenotype and contractility. *Am J Physiol Cell Physiol* **300**, C176-86 (2011).

76. Doyle, A.J. *et al.* Mutations in the TGF-beta repressor SKI cause Shprintzen-Goldberg syndrome with aortic aneurysm. *Nature genetics* **44**, 1249-54 (2012).

77. Villanueva, C.J. *et al.* TLE3 is a dual-function transcriptional coregulator of adipogenesis. *Cell metabolism* **13**, 413-27 (2011).

78. Farin, H.F. *et al.* Transcriptional repression by the T-box proteins Tbx18 and Tbx15 depends on Groucho corepressors. *J Biol Chem* **282**, 25748-59 (2007).

79. Ruiz-Larranaga, O. *et al.* Genetic variants associated with rheumatoid arthritis patients and serotypes in European populations. *Clin Exp Rheumatol* **34**, 236-41 (2016).

80. LeWinter, M.M. & Granzier, H. Cardiac titin: a multifunctional giant. *Circulation* **121**, 2137-45 (2010).

81. Bang, M.L. *et al.* The complete gene sequence of titin, expression of an unusual approximately 700-kDa titin isoform, and its interaction with obscurin identify a novel Z-line to I-band linking system. *Circ Res* **89**, 1065-72 (2001).

82. Herman, D.S. *et al.* Truncations of titin causing dilated cardiomyopathy. *The New England journal of medicine* **366**, 619-28 (2012).

83. Taylor, M. *et al.* Genetic variation in titin in arrhythmogenic right ventricular cardiomyopathy-overlap syndromes. *Circulation* **124**, 876-85 (2011).

84. Hamdani, N. *et al.* Crucial role for Ca2(+)/calmodulin-dependent protein kinase-II in regulating diastolic stress of normal and failing hearts via titin phosphorylation. *Circulation research* **112**, 664-74 (2013).

85. Stratton, M.M., Chao, L.H., Schulman, H. & Kuriyan, J. Structural studies on the regulation of Ca2+/calmodulin dependent protein kinase II. *Curr Opin Struct Biol* **23**, 292-301 (2013).

86. Hoch, B., Meyer, R., Hetzer, R., Krause, E.G. & Karczewski, P. Identification and expression of delta-isoforms of the multifunctional Ca2+/calmodulin-dependent protein kinase in failing and nonfailing human myocardium. *Circ Res* **84**, 713-21 (1999).

87. Alyonycheva, T., Cohen-Gould, L., Siewert, C., Fischman, D.A. & Mikawa, T. Skeletal muscle-specific myosin binding protein-H is expressed in Purkinje fibers of the cardiac conduction system. *Circulation research* **80**, 665-72 (1997).

88. Mouton, J., Loos, B., Moolman-Smook, J.C. & Kinnear, C.J. Ascribing novel functions to the sarcomeric protein, myosin binding protein H (MyBPH) in cardiac sarcomere contraction. *Exp Cell Res* **331**, 338-51 (2015).

89. Fukuzawa, A. *et al.* Interactions with titin and myomesin target obscurin and obscurin-like 1 to the M-band: implications for hereditary myopathies. *Journal of cell science* **121**, 1841-51 (2008).

90. Posch, M.G. *et al.* Cardiac alpha-myosin (MYH6) is the predominant sarcomeric disease gene for familial atrial septal defects. *PloS one* **6**, e28872 (2011).

91. Holm, H. *et al.* A rare variant in MYH6 is associated with high risk of sick sinus syndrome. *Nat Genet* **43**, 316-20 (2011).

92. Eijgelsheim, M. *et al.* Genome-wide association analysis identifies multiple loci related to resting heart rate. *Human molecular genetics* **19**, 3885-94 (2010).

93. Lai-Cheong, J.E., Parsons, M. & McGrath, J.A. The role of kindlins in cell biology and relevance to human disease. *Int J Biochem Cell Biol* **42**, 595-603 (2010).

94. Dowling, J.J. *et al.* Kindlin-2 is an essential component of intercalated discs and is required for vertebrate cardiac structure and function. *Circulation research* **102**, 423-31 (2008).

95. Catterson, J.H., Heck, M.M. & Hartley, P.S. Fermitins, the orthologs of mammalian Kindlins, regulate the development of a functional cardiac syncytium in Drosophila melanogaster. *PLoS One* **8**, e62958 (2013).

96. Ko, J.A. *et al.* PDZRN3 (LNX3, SEMCAP3) is required for the differentiation of C2C12 myoblasts into myotubes. *Journal of cell science* **119**, 5106-13 (2006).

97. Sewduth, R.N. *et al.* PDZRN3 destabilizes endothelial cell-cell junctions through a PKCzeta-containing polarity complex to increase vascular permeability. *Sci Signal* **10**(2017).

98. Williams, J.J. & Palmer, T.M. Cavin-1: caveolae-dependent signalling and cardiovascular disease. *Biochem Soc Trans* **42**, 284-8 (2014).

99. Yang, K.C. *et al.* Caveolin-1 modulates cardiac gap junction homeostasis and arrhythmogenecity by regulating cSrc tyrosine kinase. *Circ Arrhythm Electrophysiol* **7**, 701-10 (2014).

100. Pandur, P., Lasche, M., Eisenberg, L.M. & Kuhl, M. Wnt-11 activation of a non-canonical Wnt signalling pathway is required for cardiogenesis. *Nature* **418**, 636-41 (2002).

101. Cohen, E.D., Miller, M.F., Wang, Z., Moon, R.T. & Morrisey, E.E. Wnt5a and Wnt11 are essential for second heart field progenitor development. *Development* **139**, 1931-40 (2012).

102. Gros, J., Serralbo, O. & Marcelle, C. WNT11 acts as a directional cue to organize the elongation of early muscle fibres. *Nature* **457**, 589-93 (2009).

103. Gehmlich, K. *et al.* Paxillin and ponsin interact in nascent costameres of muscle cells. *J Mol Biol* **369**, 665-82 (2007).

104. Lin, W.H. *et al.* Molecular scanning of the human sorbin and SH3-domain-containing-1 (SORBS1) gene: positive association of the T228A polymorphism with obesity and type 2 diabetes. *Human molecular genetics* **10**, 1753-60 (2001).

105. Duval, A.J. *et al.* 0131: A phenotypic study of ARHGAP24 mitral valve prolapse suggests a genetic origin for fibro elastic deficiency. *Archives of Cardiovascular Diseases Supplements* **8**, 52 (2016).

106. Cappola, T.P. *et al.* Common variants in HSPB7 and FRMD4B associated with advanced heart failure. *Circulation. Cardiovascular genetics* **3**, 147-54 (2010).

107. Matkovich, S.J., Van Booven, D.J., Cappola, T.P. & Dorn, G.W., 2nd. Association of an intronic, but not any exonic, FRMD4B sequence variant and heart failure. *Clinical and translational science* **3**, 134-9 (2010).

108. Cox, G.A., Mahaffey, C.L., Nystuen, A., Letts, V.A. & Frankel, W.N. The mouse fidgetin gene defines a new role for AAA family proteins in mammalian development. *Nature genetics* **26**, 198-202 (2000).

109. Kato, N. *et al.* Meta-analysis of genome-wide association studies identifies common variants associated with blood pressure variation in east Asians. *Nature genetics* **43**, 531-8 (2011).

110. Hong, K.W. *et al.* Recapitulation of genome-wide association studies on pulse pressure and mean arterial pressure in the Korean population. *Journal of human genetics* **57**, 391-3 (2012).

111. Wang, D. *et al.* A Genetic Variant in FIGN Gene Reduces the Risk of Congenital Heart Disease in Han Chinese Populations. *Pediatr Cardiol* **38**, 1169-1174 (2017).

112. Spector, T.D. *et al.* Association between a variation in LRCH1 and knee osteoarthritis: a genome-wide single-nucleotide polymorphism association study using DNA pooling. *Arthritis and rheumatism* **54**, 524-32 (2006).

113. Shin, M.H. *et al.* Genetic association analysis of LRCH-1 for knee osteoarthritis. *Clinical and experimental rheumatology* **30**, 313 (2012).

114. Verweij, N. *et al.* Genetic determinants of P wave duration and PR segment. *Circ Cardiovasc Genet* **7**, 475-81 (2014).

115. Merrill, R.A., Plum, L.A., Kaiser, M.E. & Clagett-Dame, M. A mammalian homolog of unc-53 is regulated by all-trans retinoic acid in neuroblastoma cells and embryos. *Proc Natl Acad Sci U S A* **99**, 3422-7 (2002).

116. Takabayashi, T. *et al.* LL5beta directs the translocation of filamin A and SHIP2 to sites of phosphatidylinositol 3,4,5-triphosphate (PtdIns(3,4,5)P3) accumulation, and PtdIns(3,4,5)P3 localization is mutually modified by co-recruited SHIP2. *The Journal of biological chemistry* **285**, 16155-65 (2010).

117. Paranavitane, V., Coadwell, W.J., Eguinoa, A., Hawkins, P.T. & Stephens, L. LL5beta is a phosphatidylinositol (3,4,5)-trisphosphate sensor that can bind the cytoskeletal adaptor, gamma-filamin. *J Biol Chem* **278**, 1328-35 (2003).

118. Plovanich, M. *et al.* MICU2, a paralog of MICU1, resides within the mitochondrial uniporter complex to regulate calcium handling. *PloS one* **8**, e55785 (2013).

119. Paillard, M. *et al.* Tissue-Specific Mitochondrial Decoding of Cytoplasmic Ca2+ Signals Is Controlled by the Stoichiometry of MICU1/2 and MCU. *Cell Rep* **18**, 2291-2300 (2017).

120. Logan, C.V. *et al.* Loss-of-function mutations in MICU1 cause a brain and muscle disorder linked to primary alterations in mitochondrial calcium signaling. *Nat Genet* **46**, 188-93 (2014).

121. Nishiwaki, Y. *et al.* The BH3-only SNARE BNip1 mediates photoreceptor apoptosis in response to vesicular fusion defects. *Developmental cell* **25**, 374-87 (2013).

122. Boyd, J.M. *et al.* Adenovirus E1B 19 kDa and Bcl-2 proteins interact with a common set of cellular proteins. *Cell* **79**, 341-51 (1994).

123. Sotoodehnia, N. *et al.* Common variants in 22 loci are associated with QRS duration and cardiac ventricular conduction. *Nature genetics* **42**, 1068-76 (2010).
