# Supplementary material for: PR interval genome-wide association meta-analysis identifies 50 loci associated with atrial and atrioventricular electrical activity
Source: Nat Commun. 2018 Jul 25;9:2904. doi: 10.1038/s41467-018-04766-9 (PMC6060178; doi:10.1038/s41467-018-04766-9)
Supplement: Supplementary file 2 — Description of Additional Supplementary Files [file 41467_2018_4766_MOESM2_ESM.pdf]

Description of Additional Supplementary Files

File Name: Supplementary Data 1
Description: Cohort information: cohort characteristics

File Name: Supplementary Data 2
Description: Cohort information: genotyping information

File Name: Supplementary Data 3
Description: Gene Ontology analysis. Gene Ontology analysis. List of significantly overrepresented Gene Ontology (GO) categories based on enrichment analysis of PR GWAS using parametric and non-parametric analyses. Categories were deemed significant if they reached an FDR < 0.01 in both methods. Total: total number of gene members for the GO category. Observed: the actual number of PR genes mapping to the given GO category. Expected: the expected number of PR genes mapping the GO category. P-value (parametric): probability of observing the actual number of PR genes within the given GO category based on the hypergeometric distribution. FDR (parametric): false discovery rate adjustment of the enrichment P-value. FDR (permutation): false discovery rate based on random permutation analysis (non-parametric).

File Name: Supplementary Data 4
Description: Non-synonymous SNPs. The predicted effect on protein function of nonsynonymous SNPs associated with PR interval. For each locus we annotated all SNPs with r2 > 0.8 with the index or non-redundant SNPs.

File Name: Supplementary Data 5
Description: Cardiac and Blood eQTL analysis. European ancestry PR index SNPs associated with gene expression from RNA-seq in left atrial appendage (n=230) or GTEx whole blood (n=369). Given the few associations identified in whole blood, we expanded to include whole blood eQTL from Illumina gene expression array (n=5311). * indicates SNP-transcript associations found in LAA that do not replicate in RAA. ** indicates SNP-transcript associations where evidence for colocalization is less strong as detailed in Table 4B below.

File Name: Supplementary Data 6
Description: Co-localization of GWAS and eQTL associations. Our aim for identifying colocalizing genetic variants that jointly affect both molecular expression and the PR phenotype is to provide intuition regarding the candidate gene that may play a role in atrial conduction. In any given locus, we now identify a candidate gene from eQTL data (shaded in gray) if it meets the following three criteria: (1) the SNP-transcript association in LAA is significant at a genome-wide q-value 0.90) OR there

File Name: Supplementary Data 7
Description: Enrichment of PR interval genes in human diseases. List of significantly over-represented human disorders based on enrichment analysis of PR GWAS, using parametric and non-parametric methods. Categories were deemed significant if they reached an FDR < 0.01 in both approaches. Total: total number of gene members for the given disease. Observed: the actual

number of PR genes mapping to the given disease. Expected: the expected number of PR genes mapping the given disease. P-value (parametric): probability of observing the actual number of PR genes within the given GO category based on the hypergeometric distribution. FDR (parametric): false discovery rate adjustment of the enrichment P-value. FDR (permutation): false discovery rate based on random permutation analysis (non-parametric).

File Name: Supplementary Data 8
Description: Examination of PR SNPs in African-ancestry PR, as well as Europeanancestry QRS, RR, and AF.

File Name: Supplementary Data 9
Description: Examination in PR of index SNPs from AF, QRS, RR/HR Europeanancestry GWAS.
